# IL-1β and TNFα Differentially Influence NF-κB Activity and FasL-Induced Apoptosis in Primary Murine Hepatocytes During LPS-Induced Inflammation

**DOI:** 10.3389/fphys.2019.00117

**Published:** 2019-02-20

**Authors:** Julia Rex, Anna Lutz, Laura E. Faletti, Ute Albrecht, Maria Thomas, Johannes G. Bode, Christoph Borner, Oliver Sawodny, Irmgard Merfort

**Affiliations:** ^1^Institute for System Dynamics, University of Stuttgart, Stuttgart, Germany; ^2^Department of Pharmaceutical Biology and Biotechnology, Albert Ludwigs University Freiburg, Freiburg, Germany; ^3^Institute of Molecular Medicine and Cell Research, Albert Ludwigs University Freiburg, Freiburg, Germany; ^4^Clinic of Gastroenterology, Hepatology and Infection Diseases, Heinrich-Heine-University, Duesseldorf, Germany; ^5^Dr. Margarete Fischer-Bosch Institute of Clinical Pharmacology, Stuttgart and University of Tuebingen, Tuebingen, Germany; ^6^Spemann Graduate School of Biology and Medicine, Albert Ludwigs University Freiburg, Freiburg, Germany; ^7^BIOSS Centre for Biological Signaling Studies, Albert Ludwigs University Freiburg, Freiburg, Germany

**Keywords:** primary murine hepatocytes, macrophages, signaling, apoptosis, LPS-induced inflammation

## Abstract

Macrophage-derived cytokines largely influence the behavior of hepatocytes during an inflammatory response. We previously reported that both TNFα and IL-1β, which are released by macrophages upon LPS stimulation, affect Fas ligand (FasL)-induced apoptotic signaling. Whereas TNFα preincubation leads to elevated levels of caspase-3 activity and cell death, pretreatment with IL-1β induces increased caspase-3 activity but keeps cells alive. We now report that IL-1β and TNFα differentially influence NF-κB activity resulting in a differential upregulation of target genes, which may contribute to the distinct effects on cell viability. A reduced NF-κB activation model was established to further investigate the molecular mechanisms which determine the distinct cell fate decisions after IL-1β and TNFα stimulation. To study this aspect in a more physiological setting, we used supernatants from LPS-stimulated bone marrow-derived macrophages (BMDMs). The treatment of hepatocytes with the BMDM supernatant, which contains both IL-1β and TNFα, sensitized to FasL-induced caspase-3 activation and cell death. However, when TNFα action was blocked by neutralizing antibodies, cell viability after stimulation with the BMDM supernatant and FasL increased as compared to single FasL stimulation. This indicates the important role of TNFα in the sensitization of apoptosis in hepatocytes. These results give first insights into the complex interplay between macrophages and hepatocytes which may influence life/death decisions of hepatocytes during an inflammatory reaction of the liver in response to a bacterial infection.

## 1. Introduction

Liver diseases represent a major burden on health care in the European Union. Approximately 29 million people suffer from chronic liver diseases. The end-stage, liver cirrhosis with organ transplantation as single treatment option, accounts for 170,000 deaths per year (Blachier et al., 2013). Pathogenesis of most liver diseases is associated with sustained inflammation, causing enhanced cell death of hepatocytes and, finally, leading to chronic liver diseases (Malhi and Gores, 2008). Alcohol consumption, for example, increases permeability of the intestinal epithelial barrier resulting in the translocation of bacterial products such as lipopolysaccharide (LPS) from the intestinal lumen to surrounding lymph nodes and the liver. In the liver, LPS leads to the activation of Kupffer cells, the liver resident macrophages, via stimulation of the Toll-like receptor 4 (TLR4) and the induction of an inflammatory response contributing to the progression of alcoholic liver disease (Seki and Schnabl, 2012). Among many cytokines and chemokines, interleukin 1 beta (IL-1β) and tumor necrosis factor alpha (TNFα) are the most prominent pro-inflammatory cytokines released by LPS-activated macrophages (Tacke et al., 2009; Bode et al., 2012; Rex et al., 2016). Moreover, they have both been reported to exert cell death protective and promoting effects dependent on the cell type and the environmental conditions (Takehara et al., 1999; Malhi and Gores, 2008; Verma and Datta, 2010; Szabo and Csak, 2012). This is why we concentrated our work on these two cytokines.

As previously reported, TNFα sensitizes primary murine hepatocytes to Fas ligand (FasL)-induced caspase-3/7 activation and apoptosis (Schmich et al., 2011). Normally, TNFα signaling does not lead to cell death in hepatocytes (Varfolomeev and Ashkenazi, 2004) due to the inhibition of the caspase-8 containing TNF receptor complex II by the FADD-like apoptosis regulator (c-FLIP) as well as the induction of pro-survival pathways by activation of the transcription factor NF-κB (Irmler et al., 1997; Karin and Lin, 2002). Under certain circumstances, such as low c-FLIP levels or blockage of the NF-κB signaling pathway, TNFα can however trigger apoptotic signaling via caspase-8 activation in complex II (Micheau and Tschopp, 2003). TNFα also activates the c-Jun N-terminal kinase (JNK1/2) leading to the phosphorylation of the apoptosis facilitator Bim that is subsequently sequestered by the anti-apoptotic B cell leukemia/lymphoma 2 (Bcl-2) protein (Schmich et al., 2011; Geissler et al., 2013). Stimulation with FasL and generation of the truncated version of the BH3 interacting domain death agonist (tBid) by activated caspase-8 additionally depletes the anti-apoptotic Bcl-2 pool rendering hepatocytes more susceptible to caspase-3/7 activation and cell death (Schlatter et al., 2011; Schmich et al., 2011). In contrast to TNFα, IL-1β has been reported to protect mice from FasL-induced apoptosis (Takehara et al., 1999). We observed that IL-1β sensitizes hepatocytes to FasL-induced caspase-3/7 activation in a JNK/Bim- and Bid-dependent manner comparable to TNFα, but partially protects from cell death (Lutz et al., 2014). Surprisingly, increased caspase-3/7 activity after IL-1β and FasL stimulation did not result in the cleavage of the poly (ADP-ribose) polymerase (PARP) explaining why the cells did not die. The protection from FasL-induced cell death was associated with increased NF-κB DNA binding and the transcriptional upregulation of the caspase-8 inhibitor A20. The seemingly contradictious occurrence of increased caspase-3/7 activity and cell viability was further investigated by mathematical modeling, which revealed different hepatocyte subpopulations. While a fraction of cells survived the IL-1β/FasL co-treatment, others died via the type I or the type II apoptosis signaling pathway. This was dependent on a heterogeneous distribution of Bcl-2 proteins and variations in Fas signaling among the cell population. Therefore, IL-1β exerts two effects on the life-death balance in hepatocytes: It shifts hepatocytes to a mitochondrial type II apoptosis and increased caspase-3/7 activity following Fas activation and it activates NF-κB and induces upregulation of anti-apoptotic proteins, such as A20 that negatively regulates caspase-8 activation. Obviously, in the end more cells are able to escape apoptosis induction following IL-1β and FasL stimulation as compared to FasL alone.

NF-κB dimers are held inactive in the cytosol by binding to their inhibitors, the IκB proteins. Stimulation with either IL-1β or TNFα activates the IκB kinase (IKK) complex which then mediates phosphorylation, ubiquitination and degradation of IκBs allowing translocation of the free NF-κB dimer into the nucleus to initiate transcription (Karin and Ben-Neriah, 2000). The most prominent NF-κB dimer is the heterodimer containing the p50 and p65 subunits (Wang and Baldwin, 1998; Tak and Firestein, 2001) and we refer to this dimer whenever stating NF-κB hereafter. NF-κB induces transcription of a variety of target genes involved in inflammatory responses and cell survival (Baltimore, 2011). Furthermore, NF-κB induces the expression of its own inhibitor IκBα which then binds to NF-κB dimers and triggers translocation into the cytosol (Sun et al., 1993). This time delayed autoregulatory negative feedback loop causes the observed oscillatory behavior of NF-κB activation (Nelson et al., 2004; Covert et al., 2005).

FasL binds to its cognate receptor Fas/CD95 which is constitutively expressed on the cell surface of hepatocytes and induces the apoptotic pathway (Galle et al., 1995). Receptor activation leads to the formation of the death inducing signaling complex (DISC) and activation of caspase-8 (Hughes et al., 2009; Kallenberger et al., 2014). Processed caspase-8 can either directly activate the effector caspase-3 (type I pathway) or process Bid into its truncated version tBid which induces mitochondrial outer membrane permeabilization (MOMP) and release of pro-apoptotic factors such as cytochrome c and Smac/DIABLO into the cytosol (type II pathway) (Scaffidi et al., 1998; Krammer, 2000). Cytochrome c release induces formation of the apoptosome leading to activation of caspase-9 that can further process procaspase-3 (Zou et al., 1999). Smac/DIABLO inhibits the anti-apoptotic X-linked inhibitor of apoptosis protein (XIAP) that is an inhibitor of caspase-3 and caspase-9 (Verhagen et al., 2000). Thus, the release of pro-apoptotic factors from mitochondria leads to increased caspase-3 activity. FasL has been suggested to mediate hepatic cell death in experimental models of hepatitis (Galle et al., 1995; Streetz et al., 2000) and blocking FasL signaling pathways indeed ameliorates liver disease to various degrees (Kondo et al., 1997; Ksontini et al., 1998). FasL is primarily expressed on activated T lymphocytes as well as on natural killer (NK) cells (Arase et al., 1995; Suda et al., 1995) and upregulation is associated with pathogenesis of liver diseases such as viral hepatitis or alcoholic cirrhosis (Galle et al., 1995). However, the source of FasL during hepatic injury remains unclear and seems to depend on the experimental models used. Natural killer T (NKT) cells were previously reported to be key effector cells in Concanavalin A-mediated liver damage. Unlike NK cells that kill target cells by releasing TRAIL and granzyme B, NKT cells kill hepatocytes by expressing and/or releasing FasL in this model (Takeda et al., 2000). Other studies using α-galactosylceramide (α-GalCer)-induced liver injury as a murine model for autoimmune hepatitis showed that TNFα is involved in α-GalCer-induced upregulation of FasL on NKT cells (Biburger and Tiegs, 2005). In other scenarios, FasL expression was also attributed to macrophages or hepatocytes (Tsutsui et al., 1996; Luo et al., 1997; Mita et al., 2005).

In this study, we analyzed the influence of supernatant from LPS-treated bone marrow-derived macrophages (BMDMs) on FasL-induced apoptosis or survival of primary mouse hepatocytes under more physiological conditions. We show that TNFα mediates the apoptosis sensitization effect of the supernatant while IL-1β is more death protective. This is partly due to the fact that IL-1β and TNFα activate NF-κB differently. Surprisingly, the supernatant from unstimulated BMDMs protects from FasL-induced caspase-3/7 activation.

## 2. Results

### 2.1. IL-1β and TNFα Differentially Influence NF-κB Target Gene Expression

**Figure 1:**
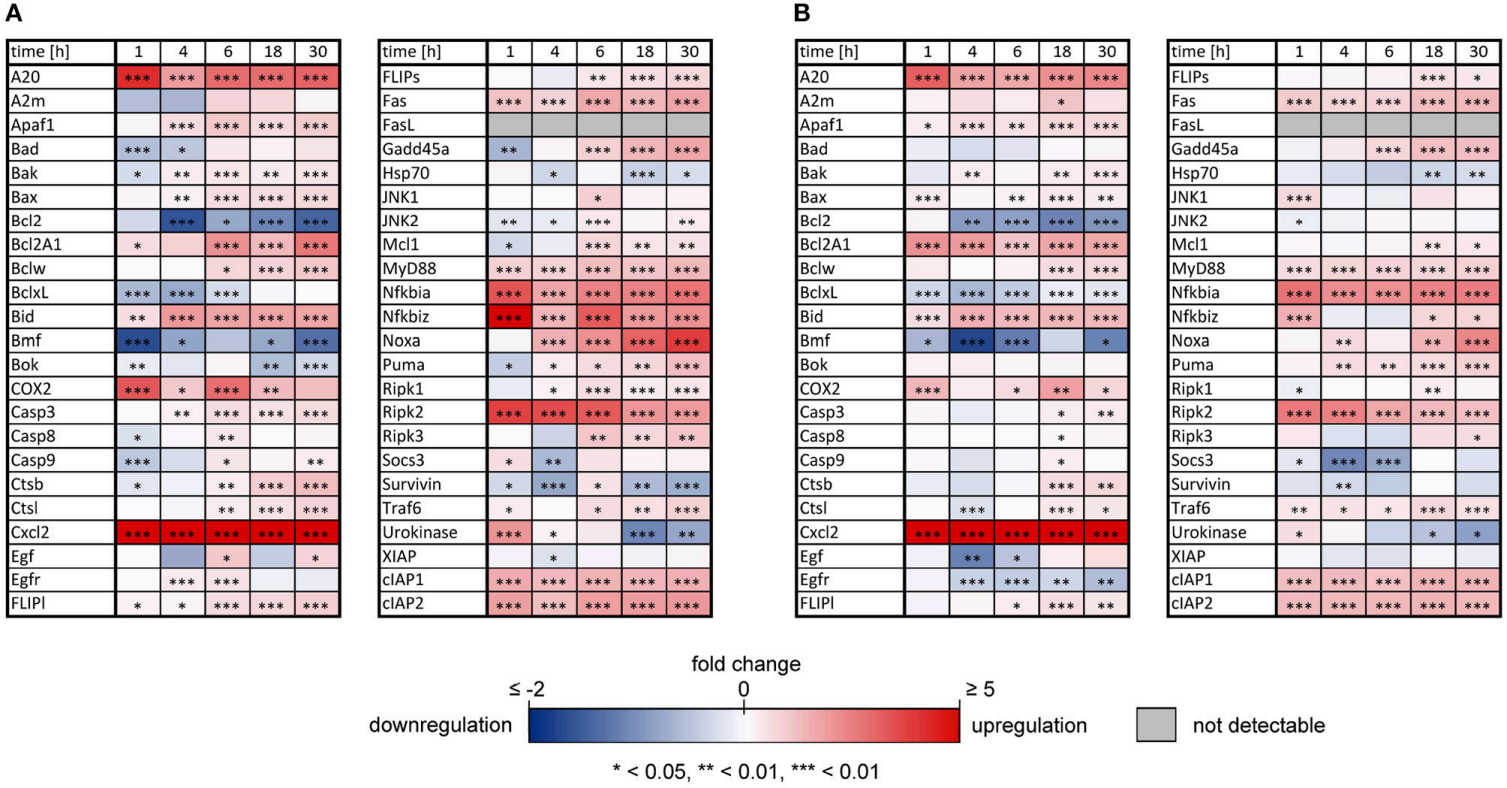
Changes of mRNAs of apoptotic and inflammatory genes after IL-1β or TNFα stimulation of hepatocytes. Gene expression pattern of primary murine hepatocytes stimulated with **(A)** IL-1β (20 ng/ml) and **(B)** TNFα (25 ng/ml) for 1, 4, 6, 18, and 30 h measured via the high-throughput Taqman® Fluidigm system. Data are analyzed using the ddCT method, normalized to untreated controls and represent 4 independent experiments. Genes marked in red and blue represent upregulated and downregulated genes, respectively (*p < 0.05, **p < 0.01, ***p < 0.001).

As previously reported, both IL-1β and TNFα sensitized primary murine hepatocytes to FasL-induced caspase-3/7 activation (Schlatter et al., 2011; Schmich et al., 2011; Lutz et al., 2014). However, while TNFα triggered increased apoptosis (Schmich et al., 2011), IL-1β partially protected from FasL-induced death, possibly via a NF-κB-dependent upregulation of survival factors such as A20, an inhibitor of caspase-8 activation (Daniel et al., 2004; Lutz et al., 2014). To uncover differences in NF-κB activity and induction of respective target genes that may be responsible for the distinct effects of these cytokines on cell viability, the mRNA levels of 46 genes involved in apoptotic and inflammatory processes were measured. For that purpose, primary murine hepatocytes were treated with IL-1β or TNFα for 1, 4, 6, 18, and 30 h and mRNA levels were determined using the high-throughput Taqman® Fluidigm Technology. Data were analyzed using the ddCT method (Livak and Schmittgen, 2001), normalized to untreated controls and results are displayed in a heat map (Figure 1).

**Figure 2:**
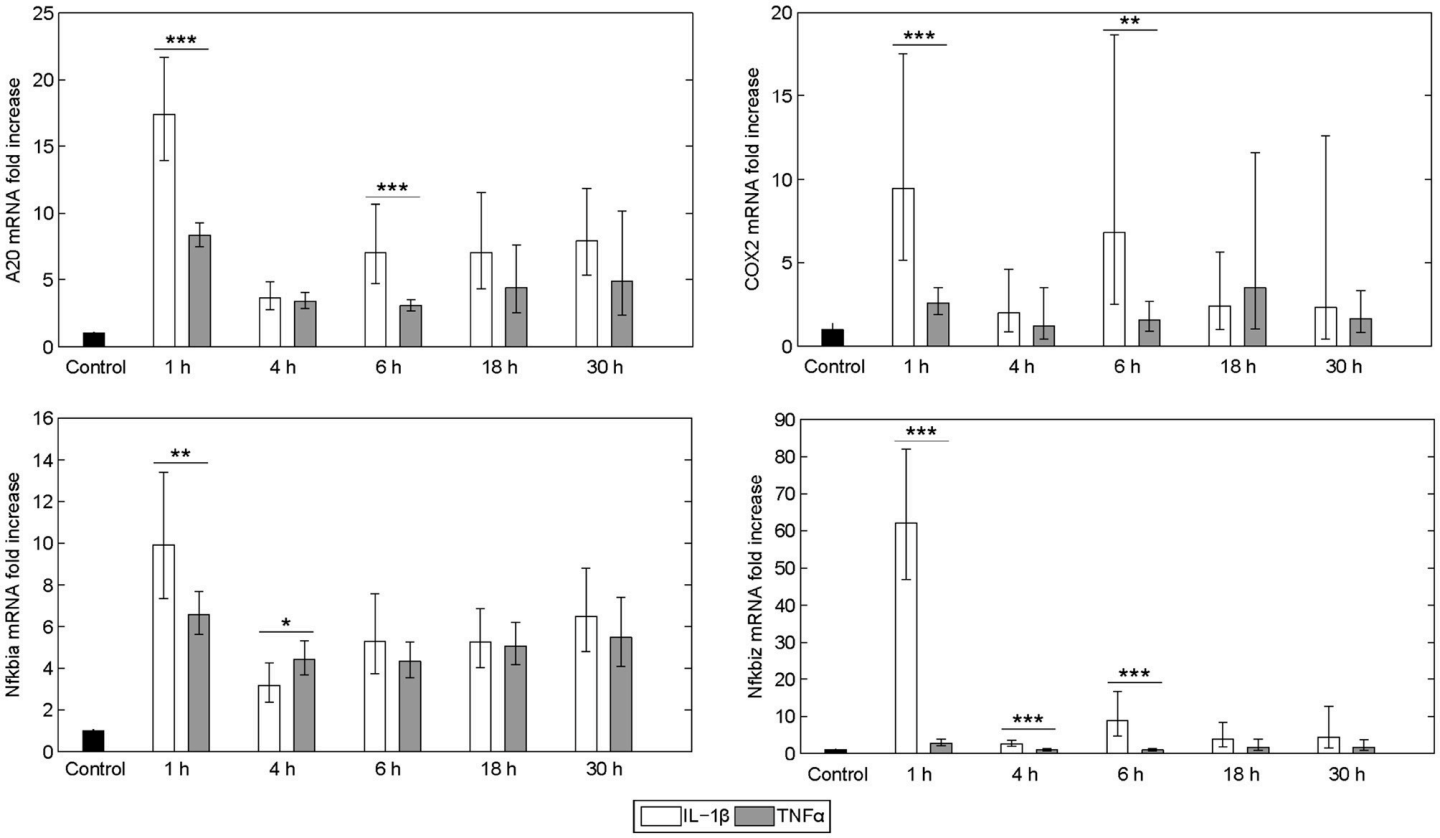
Differential gene regulation by IL-1β and TNFα. mRNA from selected genes of primary murine hepatocytes stimulated with IL-1β (20 ng/ml) or TNFα (25 ng/ml) for 0, 1, 4, 6, 18, and 30 h. Gene expression was measured via the high-throughput Taqman® Fluidigm system. Data are analyzed using the ddCT method and normalized to untreated controls. Means of 4 independent experiments ± s.d. are displayed (***p < 0.001, **p < 0.01, *p < 0.05, IL-1β vs. TNFα treated cells at the corresponding time point).

The expression pattern following stimulation with either IL-1β or TNFα appeared rather similar. mRNA of the chemokine ligand *Cxcl2* and the receptor-interacting serine-threonine kinase *Ripk2* showed the strongest upregulation. Genes involved in the NF-κB signaling pathway, i.e., the NF-κB inhibitors *I*κ*B*α (also named *Nfkbia*) and *I*κ*B*ζ (also named *Nfkbiz*), as well as the zinc finger protein *A20*, were highly upregulated after both stimuli, whereas the Bcl-2 family members *Bcl2A1* and *Bid*, as well as *Fas* and the cellular inhibitor of apoptosis proteins 1 and 2 (*cIAP1*/*2*) were increased to a lesser extent. Despite an apparently similar expression pattern after both treatments, we noted some important differences. The induction of several genes such as *A20, COX2, I*κ*B*α/*Nfkbia*, and *I*κ*B*ζ/*Nfkbiz* during the first hour of stimulation as well as their oscillations thereafter were more pronounced for IL-1β as compared to TNFα (Figure 2). The expression of *I*κ*B*ζ was even 62 times higher after IL-1β as compared to an upregulation of only 2.7 fold after TNFα stimulation. The Bcl-2 family members *Bcl-2, Bmf*, and *BclxL* showed the strongest downregulation after IL-1β and TNFα stimulation. Fas ligand (FasL) was not expressed at all time points after both stimuli.

### 2.2. Model-Based Investigation of NF-κB Dynamics and Cell Fate Following IL-1β and TNFα Stimulation

**Figure 3:**
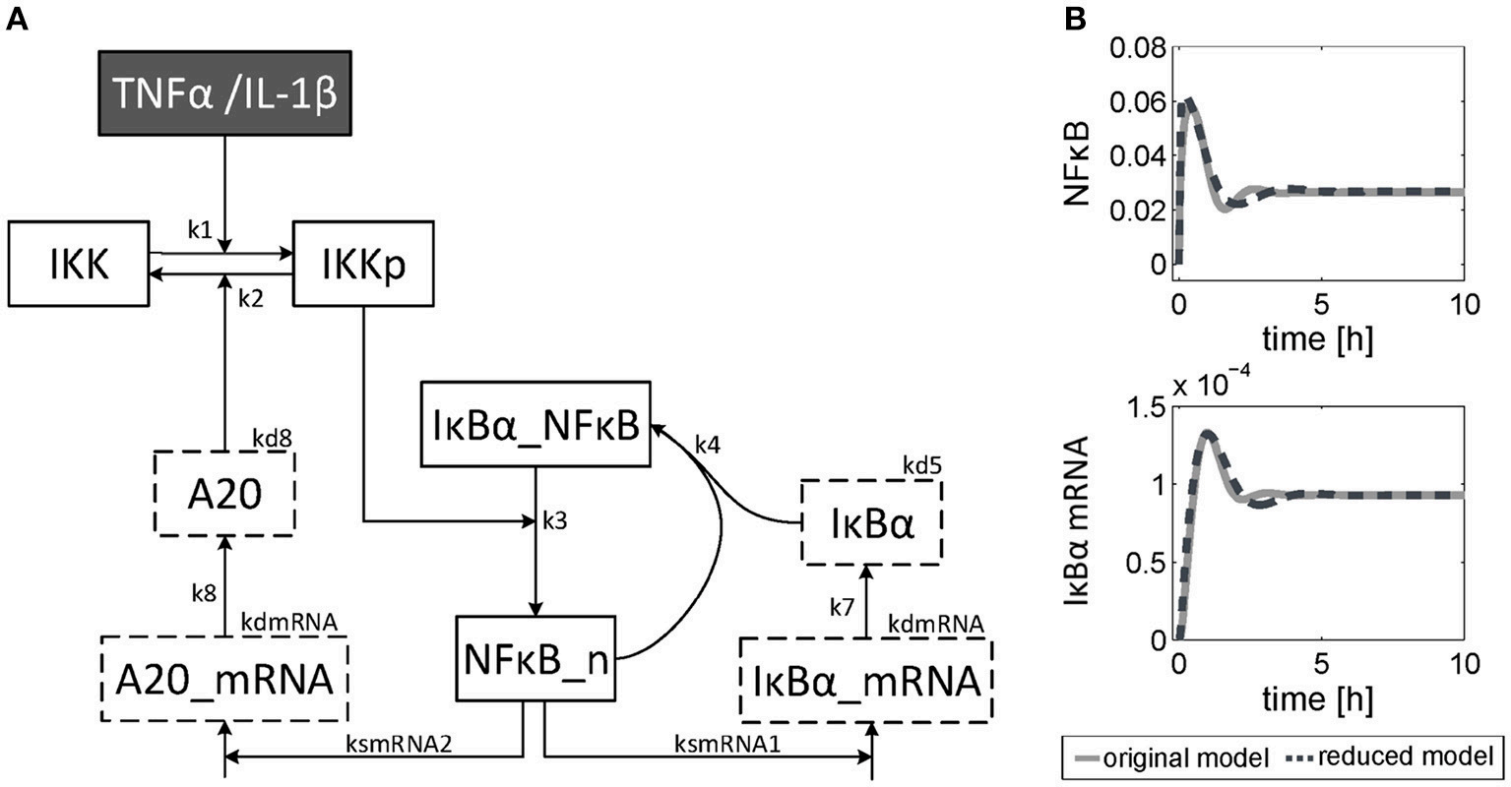
Reduced NF-κB model. **(A)** Structure of the reduced NF-κB module. The model consists of 8 species and 10 parameters. Input of the model is either TNFα or IL-1β. The model is based on ordinary differential equations and mass action kinetics. Degradation of species is indicated by boxes with dashed border. **(B)** Simulated time courses of NF-κB and IκBα mRNA of the reduced model (dashed line) compared to the original one (solid line) in response to TNFα stimulation.

**Figure 4:**
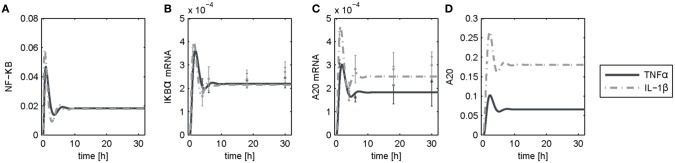
Simulation results following TNFα and IL-1β stimulation. Simulated time courses of the reduced model of NF-κB **(A)** and measured levels of mRNA **(B,C)** as well as protein **(D)** expression after stimulation with TNFα or IL-1β.

The dynamics of NF-κB have not yet been investigated in detail, although a NF-κB module has been part of our previously published models for the IL-1β/FasL (Lutz et al., 2014) and TNFα/FasL (Schlatter et al., 2011) sensitization regimens. The NF-κB model originally described by Lipniacki et al. (2004) has been integrated in our models to allow description of cytokine-mediated transcriptional effects on the FasL-induced apoptotic pathway. But the model is rather comprehensive with 14 species and 26 parameters and extensively describes the induced signaling events and complex formation between IKK, IκBα and/or NF-κB. However, for the observed effects within this study, mainly the dynamics of NF-κB activity and longer-term upregulation of NF-κB target genes are decisive. We therefore reduced the model to 8 states and 10 parameters, as described in detail in the Supplementary Material (Presentation 1). The reduced model (Figure 3A) still shows a comparable behavior to the original model regarding the aforementioned aspects (Figure 3B). Investigations revealed that a change of parameters influencing the activation of NF-κB, i.e., the parameters for the activation and deactivation of IKK (*k*_1_, *k*_2_), for A20 synthesis (*k*_*smrna*2_, k_8_) or for direct NF-κB activation (*k*_3_) mainly influence the amplitude of the first peak of NF-κB activity. By contrast, changing the parameters of the reactions which deactivate NF-κB, i.e., complex formation of NF-κB and IκBα (*k*_4_) or degradation of IκBα (*k*_*d*5_), mainly affected the frequency of NF-κB activity (Figure S1). Especially the alteration of more than one parameter such as one for activation and one for deactivation of NF-κB, e.g., *k*_3_ and *k*_4_, resulted in a more pronounced oscillatory behavior of NF-κB in response to IL-1β. Indeed, as mentioned above, *A20* mRNA is more upregulated after IL-1β than after TNFα. This difference was already confirmed on the protein level in the preceding study (Lutz et al., 2014). Accordingly, a 5-fold increase of the parameters *k*_3_ and *k*_4_ in combination with an increase of the mRNA synthesis rate of *A20* (*k*_*smrna*2_) and a 2-fold reduction of the A20 protein degradation rate (*k*_*d*8_) may well explain the different biological responses after IL-1β and TNFα stimulation. All other parameter values were identical for both treatments. The parameter values are given in Table S1 and the simulated time courses of NF-κB and its target genes as well as A20 are presented in Figure 4, time courses of all species are shown in Figure S2.

**Figure 5:**
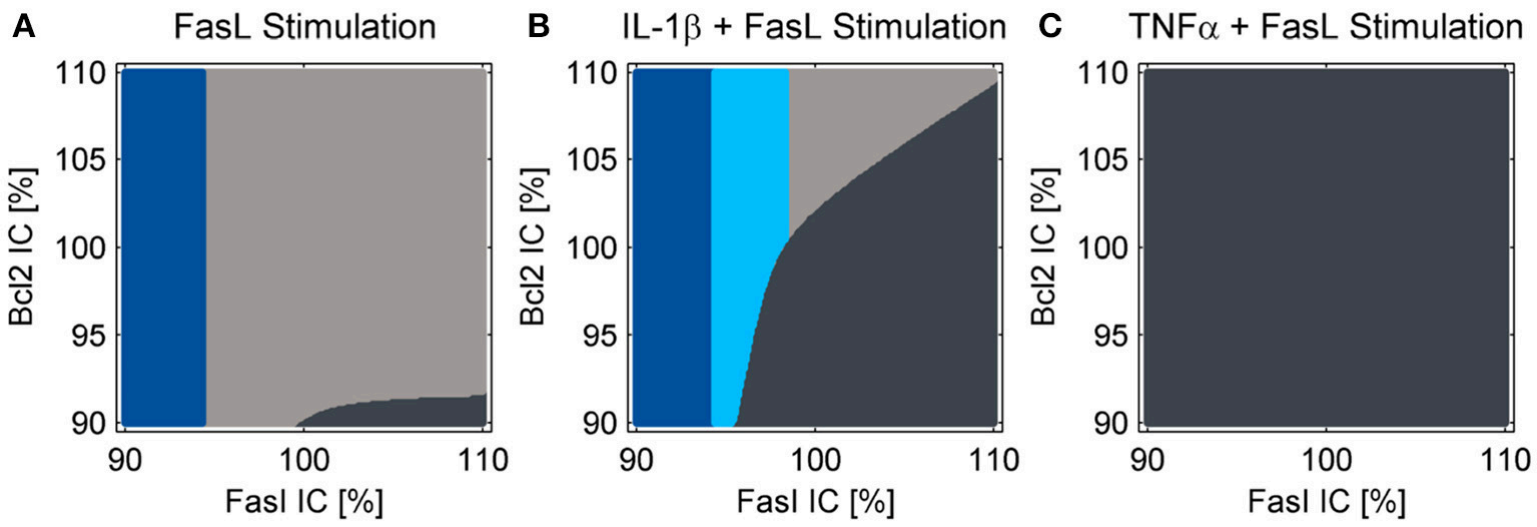
Cell fate in dependency of the initial conditions of Fas and Bcl-2. Simulations for treatment with **(A)** FasL, **(B)** IL-1β + FasL or **(C)** TNFα + FasL for different initial conditions (IC) of Fas and anti-apoptotic Bcl-2 proteins. The nominal initial conditions are 100% for both proteins and were altered ± 10%. The cells are classified as apoptotic for caspase-3 activity values above 1.5%. If cytochrome c is released during the simulation, the cells are categorized type II apoptotic and depicted in dark gray. Otherwise they are classified as type I apoptotic and illustrated in light gray. Cells with minor levels of caspase-3 activity below 1.5% are designated as survivors depicted in blue. The light blue fraction illustrates conditions for which cells survive the combined treatment with IL-1β + FasL but would die after a single FasL stimulation.

The reduced NF-κB module was implemented and the influence of increased A20 expression following IL-1β stimulation on cell fate decisions was investigated. As reported earlier (Lutz et al., 2014), a cell population does not respond homogeneously to cell death stimuli; some cells die, others can escape apoptosis induction. This could result from differences in gene expression levels among different cells. In agreement with our earlier studies, we analyzed two key molecules in the apoptotic pathway and how they influence cell fate: Fas as a representative of the death-receptor signaling pathway and Bcl-2 as a representative of the mitochondrial pathway. We analyzed the influence of small differences in protein expression by varying the initial conditions of these proteins for the simulations by 10%. The majority of cells died via the type I apoptotic pathway following FasL stimulation, but a few with high levels of Fas and low Bcl-2 expression used type II apoptosis signaling (Figure 5A). By contrast, those with low amounts of Fas survived the treatment as reported previously (Lutz et al., 2014). When IL-1β and FasL were combined two distinct effects were observed (Figure 5B). On the one hand, the preincubation with IL-1β depletes the anti-apoptotic pool of Bcl-2 proteins, rendering cells more susceptible to type II apoptotic signaling leading to MOMP. On the other hand, IL-1β induced NF-κB activation mediating pro-survival effects. When considering the upregulation of A20 which interfered with caspase-8 activation at the DISC, the IL-1β-induced protective effect (Figure 5B, light blue fraction) becomes more pronounced as compared to our previous studies (Lutz et al., 2014). In contrast, preincubation with TNFα not only favors MOMP via depletion of Bcl-2, but also directly activates caspase-8. Since A20 is less upregulated in this case, the cells treated with TNFα all die via type II apoptosis (Figure 5C).

### 2.3. Supernatant From LPS-Stimulated Macrophages Sensitizes Hepatocytes to FasL-Induced Apoptosis

**Figure 6:**
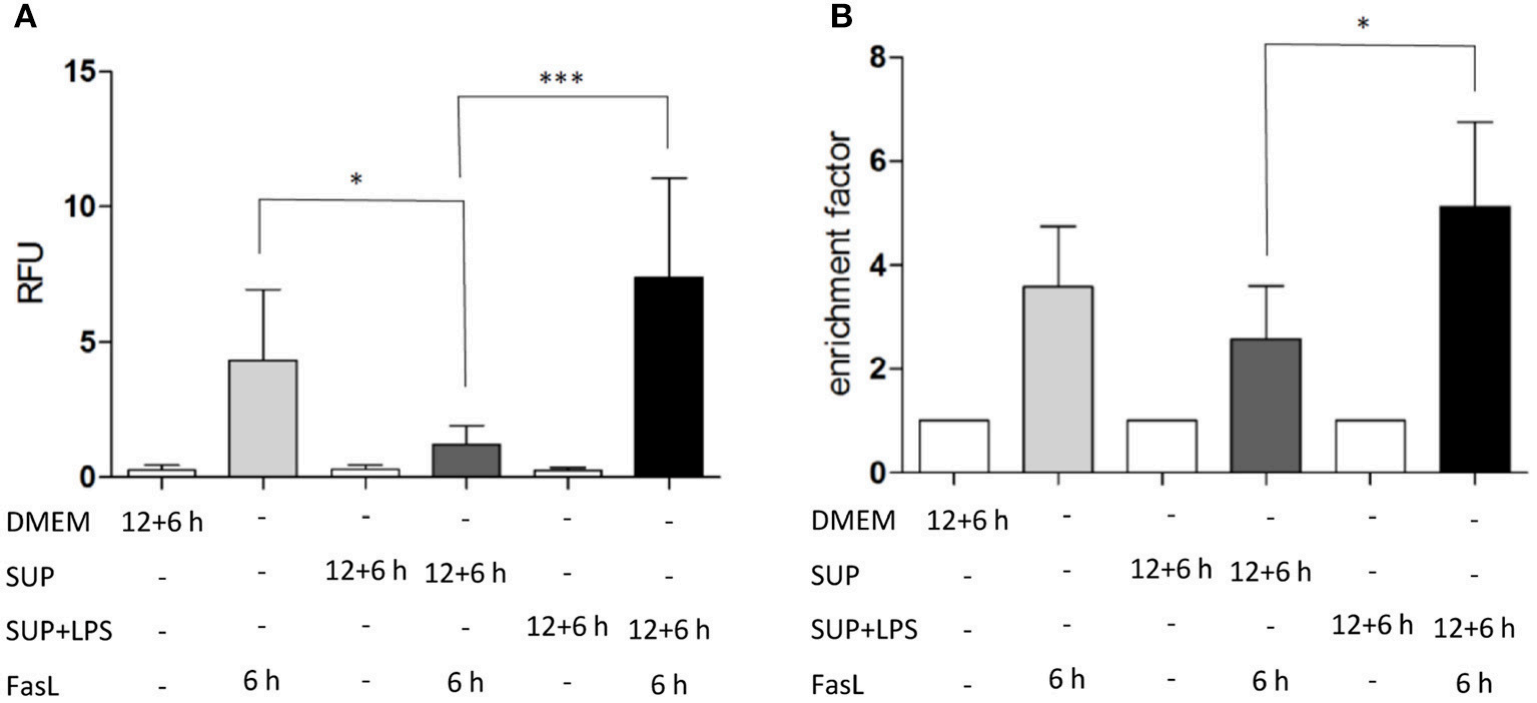
LPS-conditioned BMDM-derived supernatant sensitizes hepatocytes to FasL-induced caspase-3/7 activity and cell death. **(A)** Caspase-3/7 activity in relative fluorescent units (RFU) determined by fluorogenic DEVDase assay of hepatocytes treated with FasL (50 ng/ml) for 6 h with or without pretreatment with BMDM-derived supernatants for 12 + 6 h. Supernatants were obtained from BMDMs stimulated with LPS (100 ng/ml) for 24 h (SUP+LPS) and from untreated BMDMs (SUP). **(B)** Cell death ELISA detecting DNA fragmentation (expressed as enrichment factor) in cells treated as described above. Values are normalized to untreated controls and represent three independent experiments. Mean and standard deviation is shown (*p < 0.05, ***p < 0.001).

To study the differential sensitization effects of IL-1β and TNFα on FasL-induced apoptosis in a more physiological setting, the influence of supernatants from murine BMDMs stimulated with 100 ng/ml LPS for 24 h on apoptotic signaling in hepatocytes was investigated. Primary murine hepatocytes were cultured on collagen and, after starvation, incubated for 4 h with the same DMEM medium that was also used for BMDMs. Then, hepatocytes were preincubated with BMDM-derived supernatant conditioned with or without LPS for 12 h followed by incubation with 50 ng/ml FasL for further 6 h. Similar to the sensitizing effect of the single cytokines, a significant increase in caspase-3/7 activity (Figure 6A) and cell death (Figure 6B) was detected when using the LPS-conditioned supernatant together with FasL as compared to treatment with supernatant from untreated BMDMs in the presence of FasL. Surprisingly, the caspase-3/7 activity in hepatocytes treated with BMDM-derived supernatant without LPS stimulation and FasL (Figures 6A,B, dark gray bars) was even lower than after treatment with FasL alone (Figures 6A,B, light gray bars), indicating that untreated macrophages may secrete factors which protect against FasL-induced cell death.

### 2.4. Sensitization of Hepatocytes to FasL-Induced Apoptosis by the Supernatant From LPS-Treated Macrophages Is Mainly Mediated by TNFα

**Figure 7:**
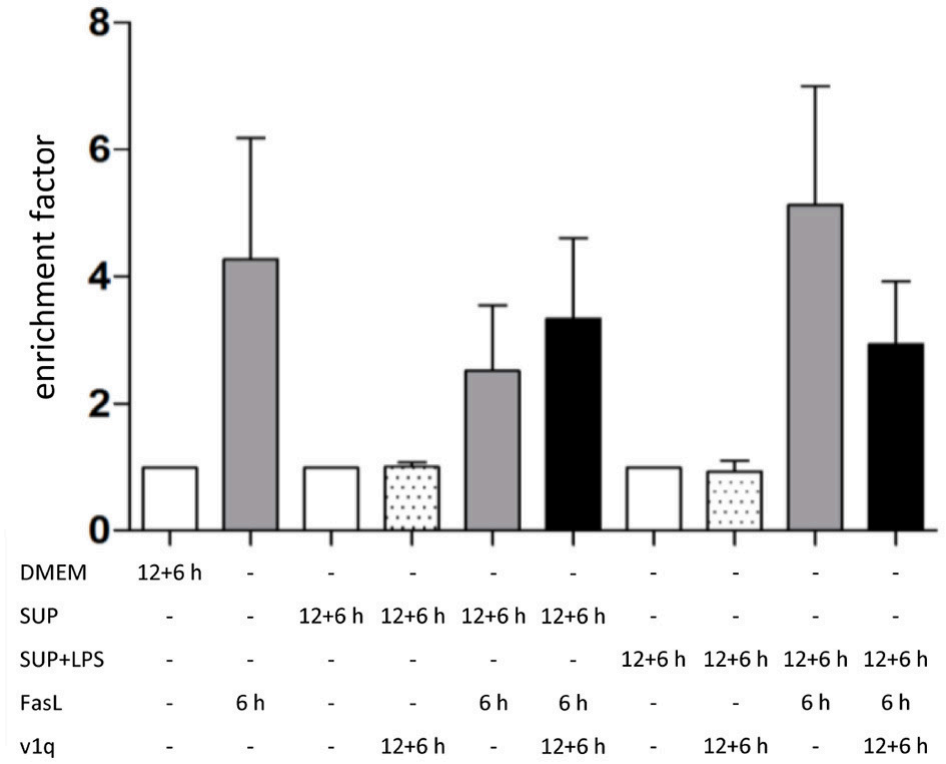
Influence of TNFα in BMDM-derived supernatants on FasL-induced apoptosis. Primary murine hepatocytes were cultured on collagen and, after starvation, preincubated with BMDM-derived supernatant conditioned with (SUP+LPS) and without (SUP) LPS (100 ng/ml) for 24 h as well as with supernatant from cultures of the v1q cell line containing TNF-neutralizing antibodies (Quantification of v1q TNF-neutralizing antibodies is described in Echtenacher et al. (1990) and Schmich et al. (2011). Cells were stimulated with FasL (50 ng/ml) for another 6 h and DNA fragmentation was measured using the cell death ELISA Kit plus. Mean value of three independent experiments with standard deviation is shown.

To investigate the role of TNFα in the apoptosis sensitization effect of BMDM-derived supernatants, hepatocytes were stimulated as described above in the absence and presence of TNF-neutralizing antibodies. Cells treated solely with BMDM-derived supernatant with and without LPS in the presence of TNF-neutralizing antibodies did not show any DNA fragmentation, as expected (Figure 7, dotted bars). Hepatocytes treated with BMDM-derived supernatant without LPS showed similar cell death rates after stimulation with FasL alone irrespective of the presence of the TNF-neutralizing antibodies. However, cells treated with LPS-conditioned BMDM-derived supernatant and FasL displayed a reduction in DNA fragmentation in the presence of the neutralizing antibodies as compared to their absence Figure 7). This finding indicates that TNFα is the cytokine secreted by macrophages which exerts the main sensitizing effect on FasL-induced apoptosis in hepatocytes. Although the data (*n* = 3) were not significant, they showed a clear tendency.

### 2.5. Supernatant From Unstimulated Macrophages Protects From FasL-Induced Caspase-3 Activation

**Figure 8:**
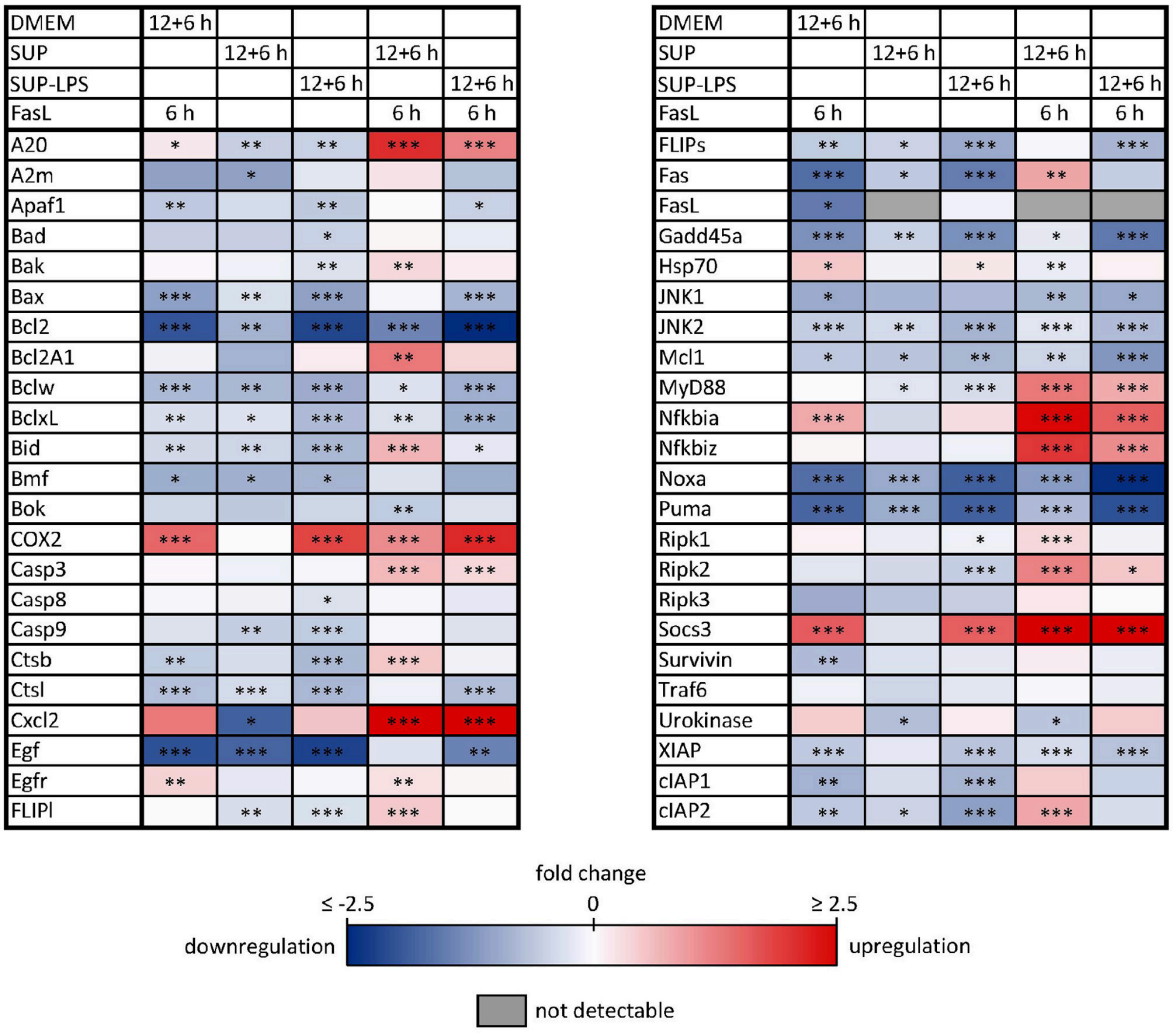
Influence of BMDM-derived supernatant on mRNA expression of apoptotic and inflammatory genes. Primary murine hepatocytes were stimulated with supernatant from murine BMDMs for 12 h that was conditioned with (SUP-LPS) and without (SUP) LPS (100 ng/ml for 24 h). Subsequently, cells were treated with FasL (50 ng/ml) for further 6 h and mRNA was analyzed with the high-throughput Taqman® Fluidigm system. Hepatocytes treated with unconditioned DMEM and FasL (50 ng/ml) served as control. Data are analyzed using the ddCT method, normalized to untreated controls (hepatocytes treated with DMEM only) and represent 4 independent experiments. mRNAs marked in red and blue are upregulated and downregulated, respectively (*p < 0.05, **p < 0.01, ***p < 0.001).

**Figure 9:**
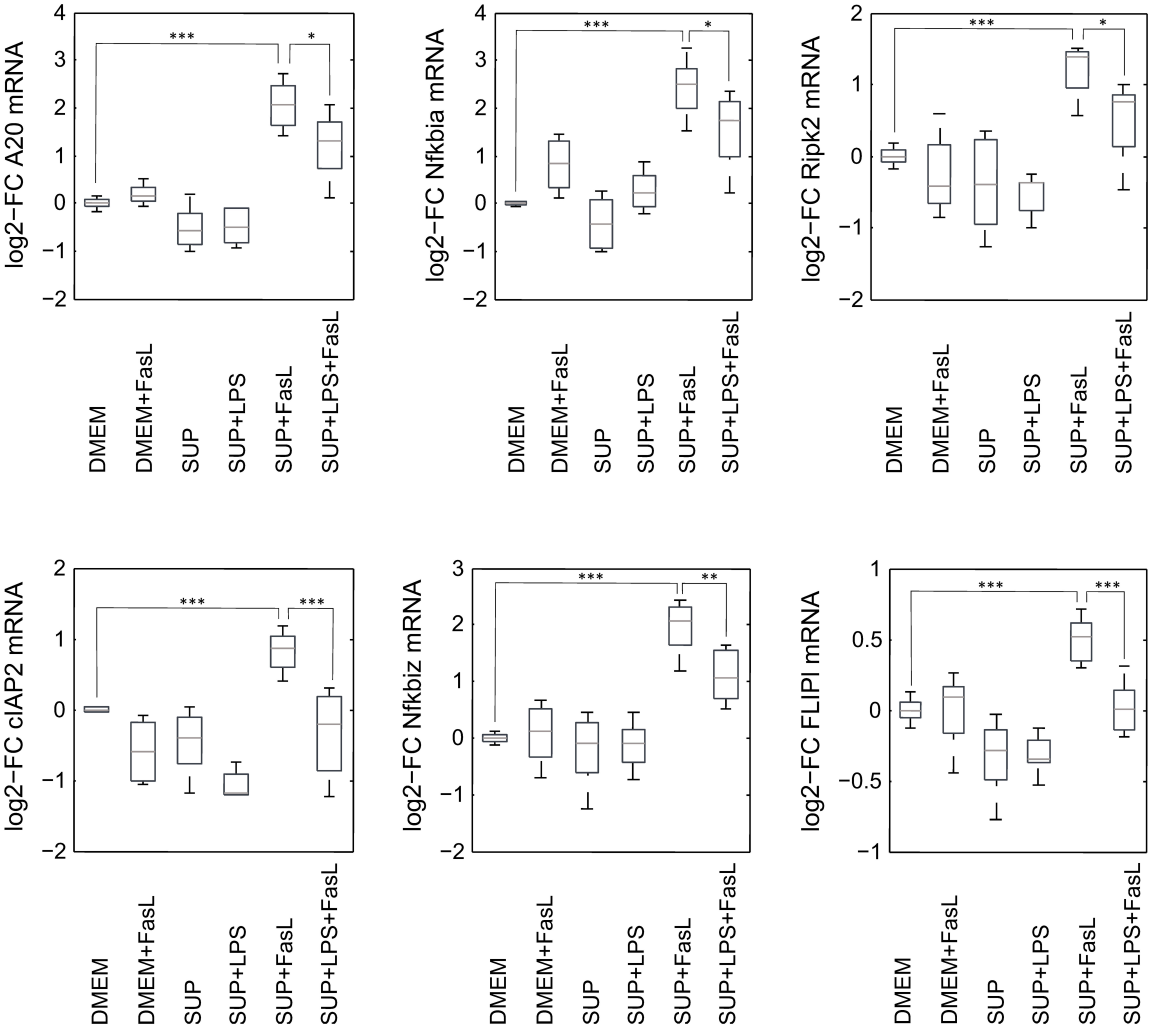
mRNA expression levels of selected genes after stimulation with BMDM supernatant and/or FasL. mRNA expression of primary murine hepatocytes stimulated with supernatant from murine BMDMs that was conditioned with (SUP+LPS/SUP+LPS+FasL) and without LPS (SUP/SUP+FasL) and/or FasL as indicated. Gene expression was measured via the high-throughput Taqman® Fluidigm system. Data are analyzed using the ddCT method, normalized to untreated controls (DMEM) and represent 4 independent experiments. Values are expressed as log2-fold change (FC) of untreated control cells and differential expression was assessed using the two-sample Student's *t*-test (*p < 0.05, **p < 0.01, ***p < 0.001).

Similar to the protective effect of IL-1β (Lutz et al., 2014), supernatants from resting BMDMs also appeared to protect from FasL-induced caspase-3/7 activation in hepatocytes (Figure 6). We therefore reinvestigated the effects of stimulation with conditioned BMDM-derived supernatant on the 46 genes involved in apoptosis and inflammation. Hepatocytes were treated with supernatant from BMDMs (18 h) that was conditioned with LPS (24 h, 100 ng/ml) and/or with FasL (6 h, 50 ng/ml) and mRNA levels were determined using the high-throughput Taqman® Fluidigm Technology. Data were analyzed using the ddCT method (Livak and Schmittgen, 2001), normalized to untreated controls (18 h DMEM) and results are displayed in a heat map (Figure 8). The strongest upregulation after most treatments is shown with the mRNAs of *COX2, Cxcl2*, and *Socs3*. These are genes typically expressed at sites of inflammation (Vane et al., 1994; Bode et al., 1999; De Filippo et al., 2013). In contrast, the mRNAs of the growth factor *Egf* as well as of the Bcl-2 proteins *Bcl-2, Noxa*, and *Puma* exhibited the strongest downregulation. Most of the other genes investigated also exhibit the tendency to reduced expression levels compared to controls. Stimulation with supernatant from resting BMDMs (Figure 8, 2nd column) abrogated the upregulation of *COX2, Cxcl2* and *Socs3* compared to the other treatments. The scenario that varies the most was the stimulation with supernatant from untreated BMDMs and FasL (Figure 8, 4th column). The inhibitors of NF-κB activation, *I*κ*b*α/*Nfkbia* and *A20*, as well as *I*κ*B*ζ/*Nfkbiz* and *Ripk2* were strongly upregulated as compared to controls and treatment with LPS-conditioned supernatant and FasL (Figure 9). In addition, mRNAs of the Bcl-2 protein *Bcl2A1* and *Bid*, cathepsin B (*Ctsb*), *FLIPl, Fas*, and *cIAP2* were upregulated by treatment with supernatant from untreated BMDMs and FasL compared to all other stimulations. Again, the regulators of FasL-mediated apoptosis, the long splice variant of c-FLIP (*FLIPl*) and *cIAP2* were significantly higher expressed after treatment with supernatant from resting BMDMs and FasL compared to controls and stimulation with LPS-conditioned supernatant and FasL (Figure 9). In summary, the supernatant from resting macrophages in combination with FasL treatment induces differential expression of NF-κB target genes which could favor the observed reduction in caspase-3/7 activation.

### 2.6. NKT Cells Are the Source of FasL in the Liver During an Inflammatory Response

**Figure 10:**
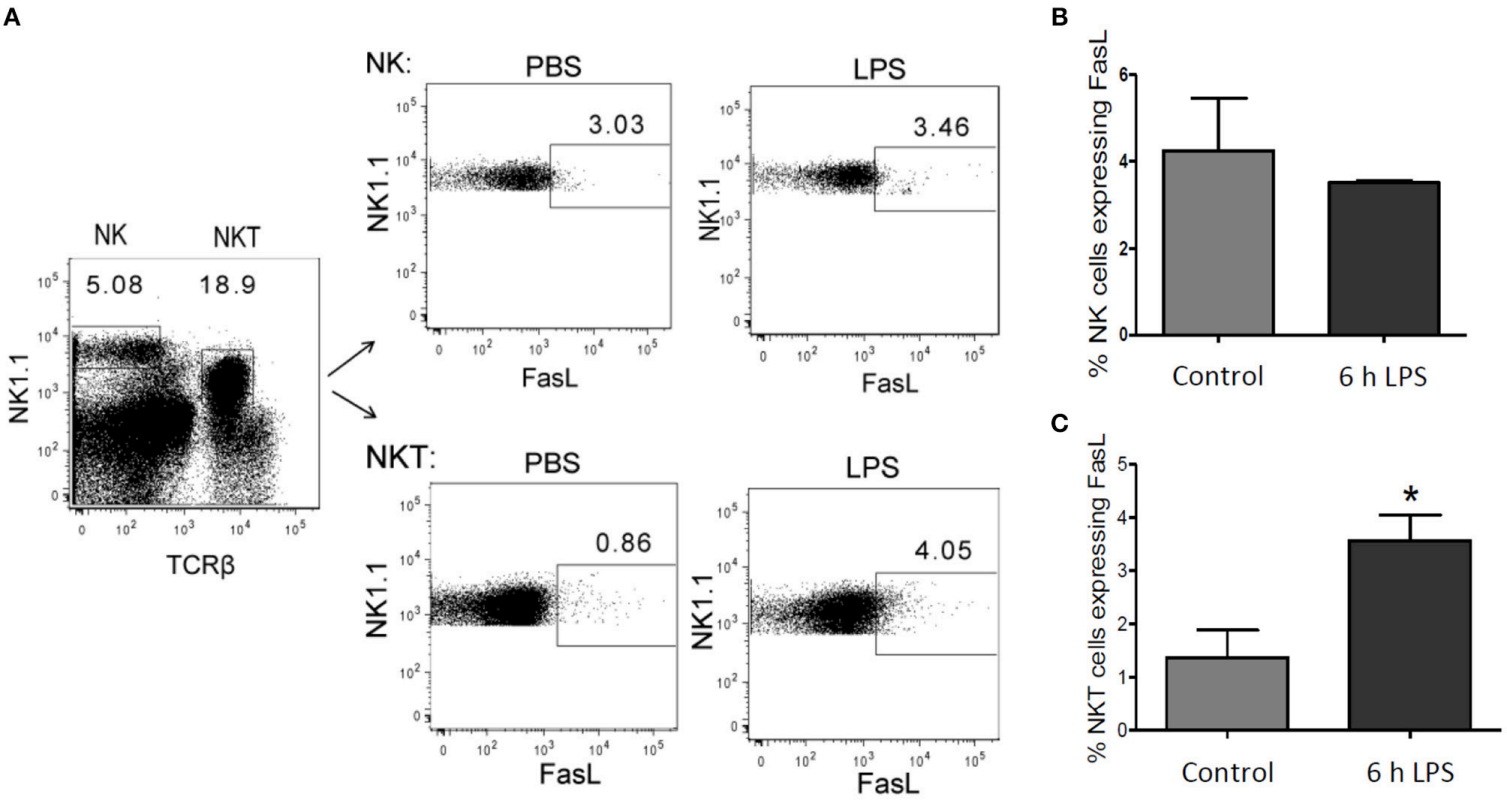
FasL expression in NK and NKT cells after LPS injection. Mice were treated with LPS or PBS for control. After 6 h the liver was perfused using collagenase to obtain a single-cell suspension. Lymphocytes were isolated using density gradient centrifugation, as described in the materials and methods section. **(A)** Representative density plots showing gate in NK (NK1.1 positive) and in NKT (NK1.1 and TCRβ double positive) cells. Number of FasL positive cells in **(B)** the NK cell- and **(C)** NKT cell-pool from LPS-treated mice (black bar) and control mice (gray bar). Mean and standard deviation is shown (*p < 0.05).

The endogenous production of FasL has been supposed to mediate hepatic cell death in the context of inflammatory disease (Galle et al., 1995; Streetz et al., 2000), but the source of FasL in the liver following LPS stimulation has remained unclear. In isolated primary murine hepatic stellate cells (HSCs) and BMDMs no LPS-mediated mRNA expression of FasL could be detected (own unpublished results). Hepatocytes also did not appear to be the source of FasL (Figure 1). Therefore, we investigated in our *in vivo* model whether NK and/or NKT cells express FasL. Mice were injected with 1 μg LPS/g of body weight and sacrificed after 6 h to obtain the NK and NKT liver cell population. Using cytometric analysis it could be demonstrated that in control mice FasL is expressed mainly on the surface of NK cells but not NKT cells. Upon LPS stimulation, however, the expression of FasL significantly increased on NKT, but not on NK cells (Figure 10).

## 3. Discussion

Pro-inflammatory cytokines are involved in various aspects of liver pathogenesis such as sustained inflammation, hepatocyte cell death as well as the chronification of liver disease (Malhi and Gores, 2008; Tacke et al., 2009). We previously reported that both IL-1β as well as TNFα sensitized primary murine hepatocytes toward FasL-induced caspase-3/7 activation (Schlatter et al., 2011; Lutz et al., 2014). While this resulted in enhanced hepatocyte apoptosis in the case of TNFα, a death protective effect was noted for IL-1β (Lutz et al., 2014). Both cytokines potently activate NF-κB (Luedde and Schwabe, 2011), which is supposed to mediate the majority of anti-apoptotic effects in hepatocytes (Tak and Firestein, 2001).

In this study, we uncovered a differential influence on the transcriptional activity of NF-κB as the possible explanation for the distinct effect of IL-1β and TNFα on hepatocyte survival. We investigated the transcriptional profile of 46 inflammatory and apoptotic NF-κB target genes after treatment of primary murine hepatocytes with these two cytokines. As expected, the gene expression pattern was qualitatively quite similar, especially regarding the inflammatory mediators (Figure 1). For example, we noted the induction of the chemokine ligand *Cxcl2*, which is known to recruit neutrophils for a hepatic inflammatory response (Krohn et al., 2009; Van Sweringen et al., 2011; Marques et al., 2012). Also high levels of *Ripk2* are expected to contribute to inflammation (Scott et al., 2010) because Ripk2 mediates innate immune signaling (Madrigal et al., 2012) and is involved in Fas-mediated NF-κB activation (Vallabhapurapu and Karin, 2009) and pro-survival signaling (Hughes et al., 2009). Similarly, mRNA of *COX2* is usually upregulated at sites of inflammation (Willoughby et al., 2000) and was reported to induce pro-survival signaling, e.g. via activation of Akt (Leng et al., 2003), and to impair apoptosis in liver cells (Fernández-Martínez et al., 2006; Casado et al., 2007). Finally, it makes sense that the mRNAs of the negative feedback inhibitors of NF-κB signaling, *I*κ*B*α and *A20* were upregulated in response to IL-1β and TNFα (Krikos et al., 1992; Sun et al., 1993). IκBα is part of the well-known NF-κB-induced autoregulatory feedback mechanism, whereas A20 interferes with both activation of NF-κB by inhibiting IKK (Skaug et al., 2011) and of caspase-8 at the DISC (Daniel et al., 2004).

Besides these similarities, we noted differences in the dynamics of how some of the NF-κB target genes were transcriptionally upregulated in response to IL-1β or TNFα. The levels of *COX2, I*κ*B*α/*Nfkbia* and *A20* mRNAs were significantly higher after IL-1β than after TNFα stimulation for various time points. Especially the first peak in gene expression after 1 h as well as the oscillations seemed more pronounced after IL-1β stimulation (Figure 2). Both COX2 and A20 have been shown to impair apoptosis in hepatocytes (Daniel et al., 2004; Fernández-Martínez et al., 2006). The biggest difference was noted for *I*κ*B*ζ/*Nfkbiz* (20-fold higher expression with IL-1β). In contrast to other IκB proteins, IκBζ localizes to the nucleus (Yamazaki et al., 2001; Totzke et al., 2006). The precise signaling roles of IκBζ have not yet been identified. IκBζ-deficient mice exhibit defective development of IL-17-producing helper T (TH17) cells and IκBζ was reported as possible transcription factor for IL-17 induction (Okamoto et al., 2010). In other studies IκBζ was described to influence NF-κB-dependent transcriptional regulation both positively and negatively (Motoyama et al., 2005). IκBζ preferably associates with p50/p50 and p65/p50 NF-κB dimers and inhibits DNA binding in the nucleus (Yamazaki et al., 2001). In this respect IκBζ may function in a pro-apoptotic manner. Indeed, transfection of IκBζ renders peritoneal macrophages more susceptible to TNFα-induced apoptosis (Yamazaki et al., 2001) and the silencing of IκBζ renders HeLa cells more resistant to apoptosis (Totzke et al., 2006). However, this pro-apoptotic property may depend on the cellular system or the type of death stimuli used. In our scenario, IκBζ is more likely to function as an anti-apoptotic factor in response to IL-1β, since this cytokine confers death protection rather than enhanced apoptosis in response to FasL treatment.

Model reduction significantly diminished the number of parameters while maintaining a very similar time course of NFκB activity and target gene expression compared to the original model. This facilitated model parametrization and allowed studying the impact of parameter variations on NF-κB activation. These investigations revealed that the amplitude and the frequency of NF-κB activity can be influenced by changing the parameter values for NF-κB activation (degradation of IκBα and liberation of NF-κB) and deactivation (reassociation of NF-κB with newly synthesized IκBα), respectively. Many posttranscriptional modifications have been described that may account different kinetics of these steps (Karin and Ben-Neriah, 2000; Perkins, 2006; Luedde and Schwabe, 2011). While the phosphorylation of two conserved serine residues of IκBα (S32/S36) target the protein for proteasomal degradation, phosphorylation of lysine residues by casein kinase II is associated with increased protein stability (DiDonato et al., 1996; Lin et al., 1996). Besides, p65 phosphorylation sites have been described to either contribute to enhanced (Zhong et al., 1997) or diminished NF-κB activity (Lawrence et al., 2005). Further posttranscriptional modifications were shown to terminate NF-κB activity (Ruland, 2011). For example, methylation of p65 at K314/K315 seemed to inhibit NF-κB activity by targeting NF-κB to proteasomal degradation (Yang et al., 2009), while acetylation of p65 prolonged NF-κB activity by preventing its binding to the inhibitory IκBα and thus nuclear export (Chen et al., 2001). All these modifications depend on the cellular stimulus and change the kinetics and dynamics of NF-κB as previously discussed (Hoffmann et al., 2002; Nelson et al., 2004; Smale, 2011). We hypothesize that IL-1β induces a more pronounced oscillatory behavior of NF-κB in hepatocytes than TNFα. Our mathematical model supports the hypothesis that differences in the oscillatory behavior of NF-κB due to distinct activation and deactivation kinetics can result in the differential upregulation of NF-κB target genes depending on the cytokine added. Whereas IL-1β induced both pro- and anti-apoptotic effects via activation of JNK and increased induction of A20, respectively, TNFα predominantly favors apoptosis induction. However, the precise regulation of the NF-κB pathway and its implication in pro-survival vs. pro-apoptotic signaling in hepatocytes during inflammatory reactions requires more detailed studies in the future.

Besides its pivotal role in inflammation, NF-κB is also described as a central player in the regulation of liver homeostasis, liver fibrosis and the development of hepatocellular carcinoma (Luedde and Schwabe, 2011; Sunami et al., 2012). The liver innate immune cell population comprises Kupffer cells, the liver-located macrophages, which are crucial for inflammatory responses (Zimmermann et al., 2012) as well as NK and NKT cells (Tacke et al., 2009). Although IL-1β and TNFα are the most important cytokines secreted by activated macrophages during an LPS-induced inflammatory response (Ulich et al., 1991; Bode et al., 2012) others such as IL-6 or IL-10 as well as the Ccl- and Cxcl-type chemokines and the type I interferon IFNβ are crucial for liver homeostasis or pathogenesis, too (Rex et al., 2016). To identify which cytokine was most important for the sensitizing effect on hepatocytes, we incubated the cells with the supernatant from LPS-treated BMDM macrophages *in vitro*. We found that the treatment of the supernatant with TNF-neutralizing antibodies tended to prevent the increase in cell death indicating that TNFα plays an important role as sensitizing mediator for apoptosis induction in hepatocytes. This is in accordance with our previous finding that macrophages secrete TNFα in much higher amounts than IL-1β (Rex et al., 2016). Therefore, TNFα should be more decisive on the fate of the cells. However, further studies are needed for final conclusions on the role of TNFα and IL-1β.

To our surprise, we observed that the supernatant from resting macrophages, i.e. without LPS conditioning, protected cells from FasL-induced caspase-3/7 activation similar to IL-1β. Investigation of the gene expression pattern revealed that FasL stimulation after preincubation with unconditioned supernatant also resulted in a differential regulation of specific NF-κB target genes such as *I*κ*B*α/*NFkbia, A20* and *I*κ*B*ζ/*Nfkbiz* (Figure 9). In addition, we noted the upregulation of *cIAP2, FLIPl*, and *Ripk2*, which are all modulators of apoptosis signaling. Ripk2 favors pro-survival signaling downstream of the DISC through induction of NF-κB activity (Festjens et al., 2007; Hughes et al., 2009). cIAP2 can directly inhibit the active forms of the caspase-3 and -7 (Roy et al., 1997) and potentiate NF-κB signaling by destabilizing IκBα (Chu et al., 1997). Both pro- and anti-apoptotic roles of FLIP_*L*_ have been reported (Chang et al., 2002). A few studies demonstrated that FLIP_*L*_ inhibits FasL-mediated apoptosis at high concentrations (Chang et al., 2002; Sharp et al., 2005) which would be in accordance with the observed effects in this study. The initial steps in apoptosis induction, formation of the DISC and the degree of caspase-8 activation has been shown to determine cell fate (Lavrik et al., 2007; Hughes et al., 2009; Kallenberger et al., 2014). It seems that after preincubation with unconditioned BMDM supernatant less apoptotic and more anti-apoptotic signaling occurs in hepatocytes after stimulation with FasL. Fas signaling usually induces the apoptotic pathway but it is also able to trigger NF-κB activation. Studies have demonstrated that the ratio of FLIP_*L*_ to caspase-8 at the DISC is decisive for apoptotic vs. pro-survival signaling (Golks et al., 2006; Fricker et al., 2010; Lavrik and Krammer, 2012). We therefore hypothesize that resting macrophages secrete protective factors that modify the balance toward pro-survival conditions such that NF-κB activation and upregulation of respective target genes prevails apoptosis induction downstream of Fas.

We finally investigated the sources of endogenous FasL in our scenario of LPS-induced inflammation since various possible sources have been reported depending on the experimental model used (Tsutsui et al., 1999; Takeda et al., 2000). NKT cells are quite abundant in the liver constituting 20–30% of the liver T cells (Bendelac et al., 1997). They have previously been implicated in liver damage during hepatitis (Takeda et al., 2000). Furthermore, FasL expression has been implicated in liver damage (Galle et al., 1995) and was associated to NK cells (Arase et al., 1995). Indeed, we could demonstrate that FasL is expressed on NK cells but strongly induced in NKT cells in our *in vivo* model of LPS-induced inflammation in mice. This suggests that endogenous production of FasL by NKT cells plays an important role in the observed hepatic cell death in inflammatory diseases (Galle et al., 1995; Streetz et al., 2000).

In summary, our study shows that it is important to investigate the aforementioned mediators and the crosstalk of pro-inflammatory cytokines released by macrophages and FasL-induced apoptotic signaling in hepatocytes in more detail. We find that macrophages modulate the hepatocytes both in the unstimulated and stimulated state. Without an inflammatory stimulus, macrophages exert a protective effect on hepatocytes, attenuate apoptosis induction and shift the balance toward pro-survival signaling. However, they sensitize hepatocytes to apoptosis induction during LPS-induced inflammation, probably to rapidly remove damaged cells.

## 4. Materials and Methods

### 4.1. Mice Strains and Primary Cell Isolation

Wild type (C57BL/6N and C57BL/6J) mice were purchased from Jackson Laboratories. Primary hepatocytes were isolated from 8 to 14 week old BL6 mice using the collagenase perfusion technique and cultivated as previously described (Klingmüller et al., 2006; Schmich et al., 2011) (see also Supplementary Material, Presentation 1). The whole study, including the isolation procedure, was approved by the animal experimental committee (ethical permission number: X-12/22D, University of Freiburg). For generation of bone marrow-derived macrophages (BMDMs) 8–12 week old male mice were sacrificed. The animal applications were reviewed and approved by the appropriate authorities and were performed in accordance with the German animal protection law (AZ: 84-02.04.2012.A175; Landesamt für Natur, Umwelt und Verbraucherschutz Nordrhein-Westfalen, Recklinghausen). All animals were handled and housed according to specific pathogen free (SPF) conditions.

### 4.2. Preparation of BMDM Supernatants

The preparation and cultivation of primary murine BMDMs has been carried out according to the standard operating procedure (SOP) as previously described (Rex et al., 2016). Summarizing, after 8 days of BMDM differentiation/cultivation (DMEM: Biochrom, Berlin, Germany; FCS (Cat.: 10099141, Lot: 769367): Invitrogen, Karlsruhe, Germany; Penicillin G/Streptomycin: Cytogen, Wetzlar, Germany), adherent cells were harvested by gentle trypsinization: Cells were washed twice with prewarmed PBS (Biochrom, Berlin, Germany) and treated with 3 ml trypsin/EDTA solution (Cytogen, Wetzlar, Germany) for approximately 5–10 min. Cells were centrifuged and adjusted in M-CSF (5 ng/ml; recombinant murine M-CSF: Peprotech, Rocky Hill, NJ, USA) containing culture medium: 1.4 × 10^6^ cells/3.5 ml per 60 mm tissue culture dish. After 6 h of cultivation, cells were stimulated with 100 ng/ml LPS (using LPS/DMEM solution; LPS from *Escherichia coli* (# L3012, Sigma-Aldrich, Munich, Germany) or were treated with the corresponding volume of Dulbecco modified Eagle medium (DMEM) as control, respectively. After 24 h, cell culture supernatant was collected under sterile conditions by centrifugation (20 min, 4°C, 5.500 rpm). Aliquots were prepared and tempered gently up to −80°C for storage.

### 4.3. Quantification of N2A FasL

Quantification of FasL in the Neuro2A supernatant was carried out as described before (Walter et al., 2008). Briefly, the human T cells Jurkat E6 were treated or untreated with Neuro2A supernatant diluted 1:4 (25%) in medium for 1 h. Defined concentrations of recombinant Fc-FasL served as the standard and apoptosis was quantified by GFP-annexin-V/PI FACS analysis.

### 4.4. Treatment of Isolated Primary Murine Hepatocytes

To investigate the influence of IL-1β and TNFα on the gene expression profile primary murine hepatocytes (1 × 10^6^ cells) were stimulated with 20 ng/ml IL-1β or 25 ng/ml TNFα (both from R&D Systems, Minneapolis, USA) for 1, 4, 6, 18, and 30 h or left untreated as control. To study the influence of macrophage-derived mediators on FasL-induced caspase-3/7 activity and cell death, hepatocytes (2 × 10^6^) were pre-incubated with the supernatant from LPS-stimulated BMDMs (SUP+LPS) or with the supernatant from untreated BMDMs (SUP) for 12 h and subsequently with 50 ng/ml FasL (generated by Neuro2A cells) for further 6 h. Additionally, hepatocytes were only stimulated with FasL for 6 h, with the supernatant from unstimulated (SUP), LPS-treated BMDMs (SUP+LPS), or the medium DMEM for 18 h as controls.

### 4.5. DEVDase Assay

The activity of the executioner caspase-3/7 in hepatocytes (1 × 10^6^) was measured by the fluorogenic DEVDase assay as previously described (Schlatter et al., 2011; Lutz et al., 2014). See also the Supplementary Material (Presentation 1).

### 4.6. Cell Death Detection ELISA

To quantify the amount of DNA fragmentation in hepatocytes (1 × 10^6^) after treatment with the different stimuli the cell death detection ELISAPLUS Kit (Roche, Mannheim, Germany) was used and performed according to the manufacturer instruction (Lutz et al., 2014) (for detailed information see the Supplementary Material (Presentation 1).

### 4.7. RNA Isolation, cDNA Synthesis and qRT-PCR

Total RNA was isolated using the RNeasyPlus Kit (Qiagen, Hilden, Germany) according to the manufacturer instruction. The quantity and purity of RNA was determined by measuring the optical density at 260 and 280 nm. 600 ng total RNA was reverse transcribed to cDNA with TaqMan Reverse Transcription Reagents (Applera GmbH, Darmstadt, Germany). For qRT-PCR the Fluidigm Biomark high throughput qPCR chip platform (Fluidigm Corporation, San Francisco, CA, USA) with pre-designed gene expression assays from Applied Biosystems was used according to the manufacturer instructions (Spurgeon et al., 2008). Data were analyzed using the ddCT method (Livak and Schmittgen, 2001) and expression values were normalized to the expression levels of the β-actin gene. All TaqMan assays are listed in the Supplementary Material in Data Sheets 1, 2 for stimulation with IL-1β/TNFα and BMDM supernatant, respectively.

### 4.8. FACS Analysis/ *in vivo* Experiments

C57BL/6 mice (8–14 weeks old) were injected i.p. with 1 μg LPS/g of body weight and sacrificed after 6 h. The livers were extracted and homogenized with a plunger rod over a 70 μm cell strainer in a 50 ml falcon. Hepatic lymphocytes were further isolated by density gradient centrifugation using 60% and 40% percoll. Cells were labeled with APC-conjugated anti-mouse NK 1.1, PerCP-conjugated anti-mouse TCRb and PE-anti mouse conjugated FasL. Background staining was determined using a PE-anti mouse IgG isotype control. All antibodies were used at a concentration of 1 μg/ml and purchased from BD Biosciences. Flow cytometry analysis was performed using FACSdiva (BD Bioscience).

### 4.9. Isolation of NK and NKT Liver Cell Populations

Single-cell suspensions were prepared from the liver by collagenase-based perfusion via the portal vein. Total liver cells were homogenized in a 40% isotonic percoll solution and slowly passed to a 60% isotonic percoll solution without mixing the layers. After centrifugation for 20 min at 900 g, the upper layer was discarded (debris and hepatocytes) and the mononuclear cells were collected from the interface. The cells were washed once with PBS (300 g, 7 min, room temperature) and then 1 ml red blood cell lysis buffer was added for 3 min. After other washing step the cells were incubated with Fc-blocking buffer for 15 min and afterwards incubated for 30 min with the following antibodies: NK1.1-APC, TCRβ-FITC and CD178-PE or Arm hamster isotype control-PE (eBioscience).

### 4.10. Statistical Analysis

Values are expressed as means ± standard deviation (s.d.). Differences in expression, caspase-3/7 activity and DNA fragmentation were assessed using the two-sample Student *t*-test. *P*-values were calculated and *p* ≤ 0.05 was considered as significant.

### 4.11. Mathematical Modeling and Simulation

The model is based on ordinary differential equations and mass action kinetics and was implemented using MATLAB R2014a. The model setup and reduction is explained and all model equations can be found in the Supplementary Material (Presentation 1).

## Author Contributions

JR, AL, LF, UA, JB, CB, and IM: conceived and designed the experiments; AL, LF, and UA: performed the experiments; JR, AL, LF, MT, and IM: analyzed the data; MT, JB, CB, OS, and IM: contributed reagents, materials, analysis tools; JR and OS: performed the mathematical modeling; JR, LF, UA, CB, and IM: wrote the paper.

### Conflict of Interest Statement

The authors declare that the research was conducted in the absence of any commercial or financial relationships that could be construed as a potential conflict of interest.
