# Supplementary material for: IL-1β and TNFα Differentially Influence NF-κB Activity and FasL-Induced Apoptosis in Primary Murine Hepatocytes During LPS-Induced Inflammation
Source: Front Physiol. 2019 Feb 20;10:117. doi: 10.3389/fphys.2019.00117 (PMC6391654; doi:10.3389/fphys.2019.00117)
Supplement: Supplementary file 3 [file Presentation_1.PDF]

Supplementary Material:
IL-1β and TNFα differentially influence NF-κB activity and FasL-induced apoptosis in primary murine hepatocytes during LPS-induced inflammation

Julia Rex *, **Anna Lutz, Laura E. Faletti, Ute Albrecht, Maria Thomas, Johannes G. Bode, Christoph Borner, Oliver Sawodny and Irmgard Merfort**
*Correspondence:
Julia Rex, Institute for System Dynamics, University of Stuttgart, Stuttgart, Germany
julia.rex@isys.uni-stuttgart.de

1 EXPERIMENTAL METHODS

1.1 Isolation and cultivation of primary mouse hepatocytes

Cells were plated on rat tail collagen I-coated tissue culture dishes in William's medium E (WME, Biochrom) supplemented with 10 % FCS, 100 nM dexamethasone, 2 mM L-glutamine and 1 %-penicillin/streptomycin solution (all reagents from Gibco). To allow hepatocytes to attach, cells were kept in a humidified atmosphere at 37°C and 5 % CO_2 for 4 h. Subsequently, the FCS containing WME was removed and replaced by serum-free WME supplemented with 100 nM dexamethasone, 2 mM L-glutamine and 1 %-penicillin/streptomycin solution. Following incubation in this medium for 4 h, hepatocytes were washed three times and further kept in starvation medium (WME supplemented with 2 mM L-glutamine and 1 %-penicillin/streptomycin solution) for 16-24 h.

1.2 DEVDase assay

The activity of the executioner caspase-3/-7 was measured by the fluorogenic DEVDase assay as previously described (Schlatter et al., 2011). Primary mouse hepatocytes (1 x 10^6) were treated with cytokines (IL-1β, TNFα) and/or in combination with FasL (generated by Neuro-2 A cells) for the indicated times. After the incubation the cells were detached, washed with PBS and centrifuged (1200 rpm at 4 °C for 3 min). The pellet was homogenized in 50 µl lysis buffer (50 mM Hepes-KOH, 2.5 mM $MgCl_2$, 2.5 mM EGTA, 12 mM DTT supplemented with the protease inhibitors 12 µg/ml aprotinin, 12 µg/ml leupeptin, 0.5 µg/ml pepstatin, 0.125 µM PMSF, 1.5 µg/ml cytochalasin B) by freeze-thaw cycles and finally centrifuged at 14000 rpm at 4 °C for 10 min. The caspase-3/-7 activity assay was performed using the DEVD-AMC (Alexis) substrate at a concentration of 200 nM. 8 µl of the protein extract were mixed with 90 µl assay buffer (50 mM Hepes-KOH, 12 mM DTT) and the DEVD-AMC substrate (Enzo Life Science, Lrrach, Germany). Subsequently the fluorescence was measured for 40 cycles and the slope was calculated. The relative fluorescence unit (RFU) was determined by the ratio of the slope and the protein concentration of each sample, measured by Bradford assay (Biorad). To compare different experiments, RFU sample values were referred to negative control (untreated cells).

1.3 Cell Death detection ELISA

Hepatocytes (1 x 10^6) were treated with the stimuli at different times. Subsequently, the cells were washed with PBS, detached and incubated in 500 µl lysis buffer for 30 min at RT. The lysate was centrifuged at 2300 rpm for 10 min and the supernatant was used to prepare a 1:10 dilution in lysis buffer. Samples and controls, 20 µl respectively, were transferred to the streptavidin-coated microplate and incubated with 80 µl of the immunoreagent (containing incubation buffer, anti-histone-biotin and anti-DNA-POD) for 2 h at 25 °C and shaking at 300 rpm. Afterwards, the wells were washed and incubated with ATBS-solution for 20 min gently shaken at 300 rpm. The reaction was stopped by adding ATBS-stop-solution and the absorbance was measured at 405 nm and 490 nm as reference wavelength. The sample values were referred to untreated control.

2 SUPPLEMENTARY FIGURES

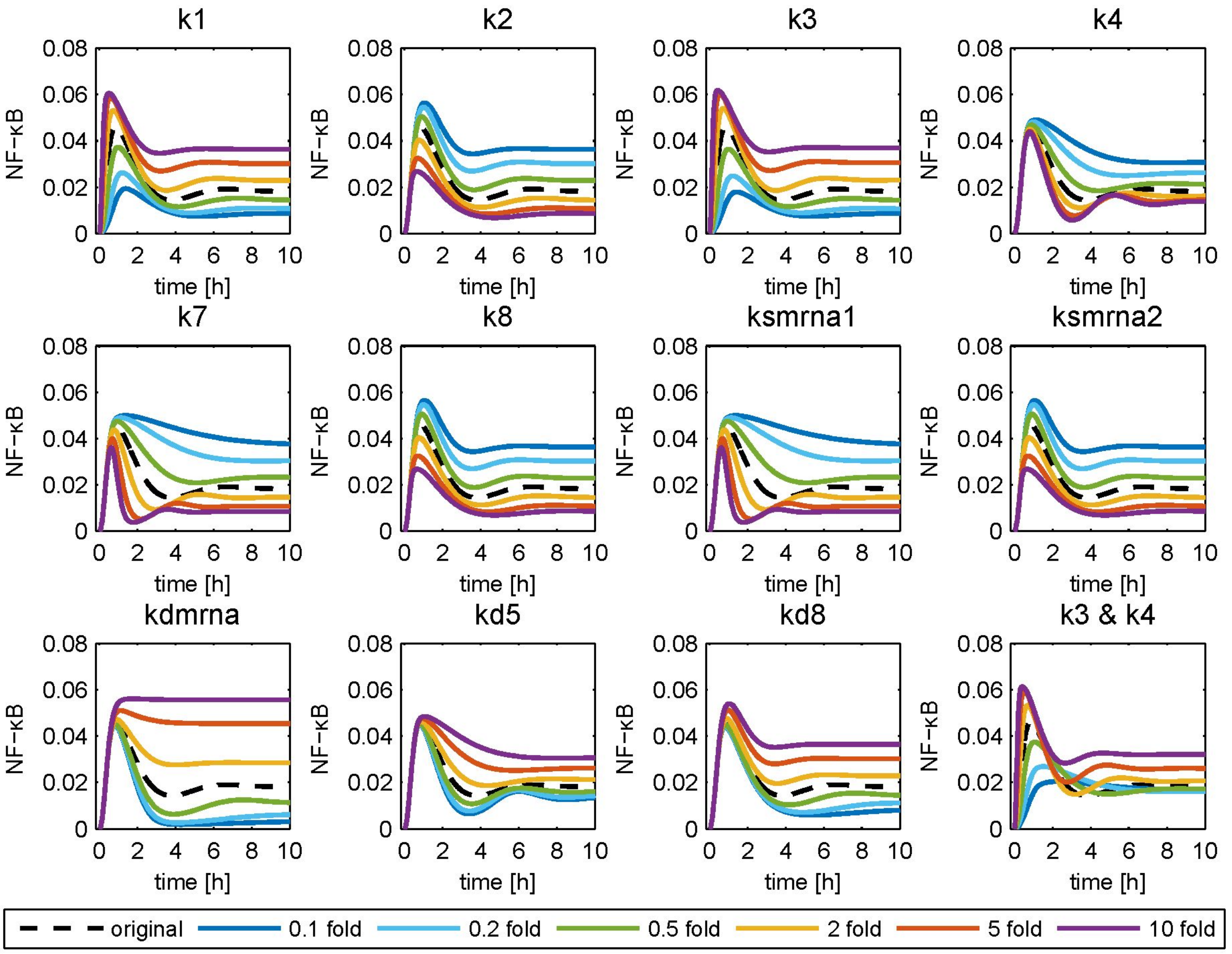

Figure S1. Influence of parameter variations on NF-κB oscillations. The values of all parameters of the NF-κB module as well as the combination of the parameters k_3 and k_4 were changed in the range of one order of magnitude and the resulting time course of NF-κB is shown.

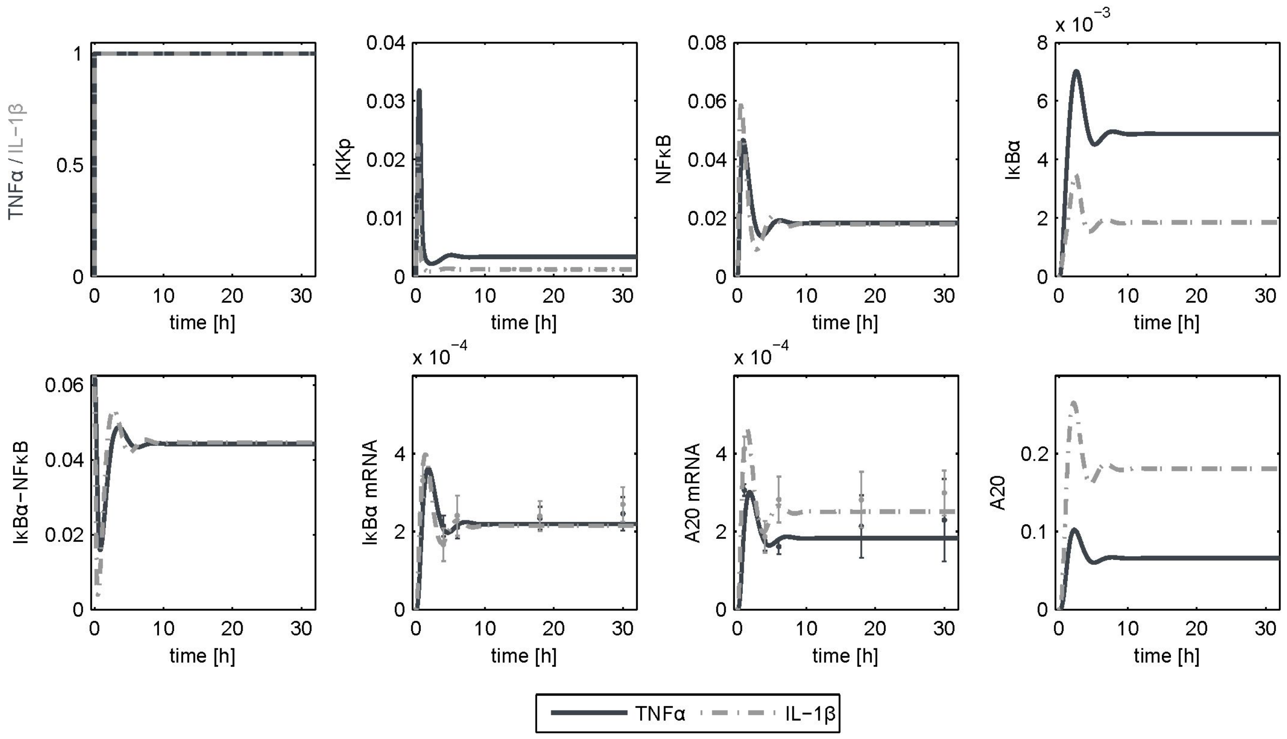

Figure S2. Simulated time courses of the NF-κB module following TNFα and IL-1β stimulation. Simulation results of the reduced NF-κB model and measured levels of mRNA expression after stimulation with TNFα or IL-1β.

3 SUPPLEMENTARY TABLES

Table S1. Parameters of the reduced NF-κB model. Notation of all parameters of the NF-κB model (Fig. 3) with a description and the according value for TNFα and IL-1β stimulation.

parameter	parameter value TNFα	IL-1β	description
k_1	0.45	0.45	activation of IKK by TNFα
k_2	400	400	inhibition of IKK by A20
k_3	90	450	activation of NF-κB by phosphorylation and degradation of IκBα
k_4	150	750	deactivation of NF-κB via binding of IκBα
k_7	150	150	IκBα protein synthesis
k_8	900	900	A20 protein synthesis
$k_{\text{smrnaI}\kappa\text{B}\alpha}$	0.012	0.012	IκBα mRNA synthesis
k_{smrnaA20}	0.010	0.014	A20 mRNA synthesis
k_{dmrna}	1	1	mRNA degradation
k_{d5}	4	4	degradation of IκBα
k_{d8}	2.5	1.25	degradation of A20

4 MODELING OF THE NF-κB MODULE

The NF-κB module that originates from the model by Lipniacki et al. (2004) was implemented in both our previous models describing the sensitizing effect of TNFα (Schlatter et al., 2011) and IL-1β (Lutz et al., 2014) on FasL-induced apoptosis. It was adopted with minor changes only as described previously (Schlatter et al., 2011) to allow description of transcriptional responses, e.g. expression of an NF-κB target gene or treatment with a transcriptional inhibitor. However, the dynamics of NF-κB activation and target gene expression in our system of primary murine hepatocytes were not investigated in detail.

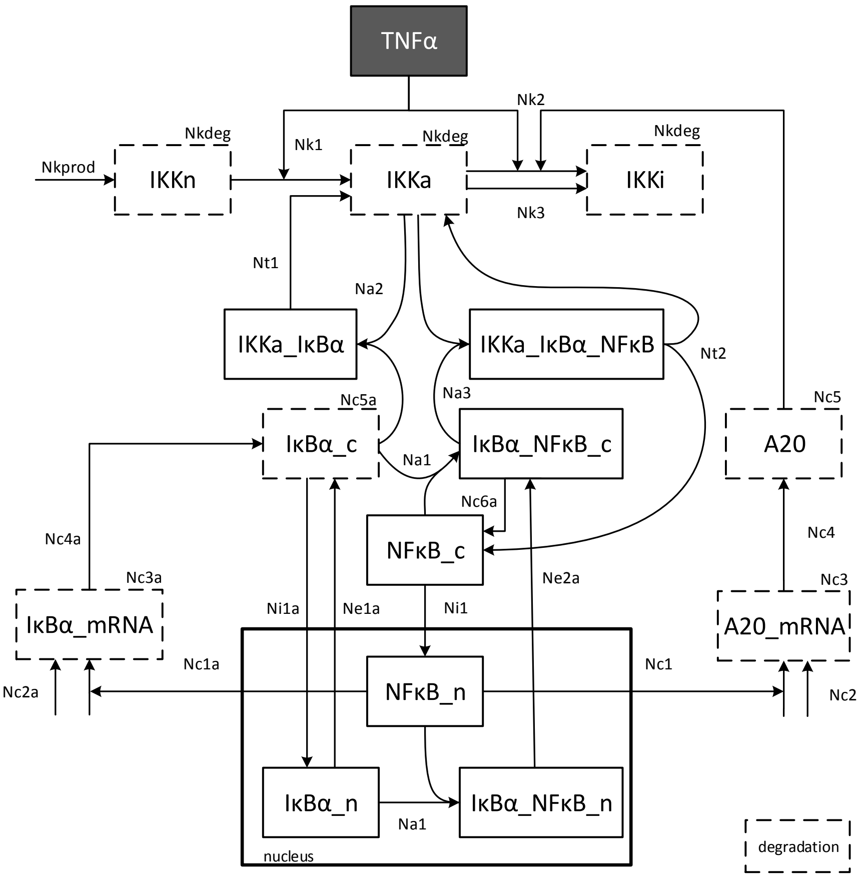

Figure S3. NF-κB module. Structure of the NF-κB module as implemented in the TNFα/FasL apoptosis sensitization model by Schlatter et al. (2011). The model has 14 species, 26 parameters and 2 compartments (cytosol and nucleus) with a volume ratio $V_{Cytosol} : V_{Nucleus} = 3$. Input of the model is TNFα. The model is based on ordinary differential equations (ODEs) and mass action kinetics. Degradation of species is indicated by boxes with dashed borders.

The NF-κB module has 14 species and 26 parameters and is illustrated in Figure S3. Simulation results are depicted in Figure S4. The parameter values are given in Table S2. Cytosol and nucleus were modeled as two separate compartments with a volume ratio of

$$k_V = \frac{V_{Cytosol}}{V_{Nucleus}} = 3. \tag{S1}$$

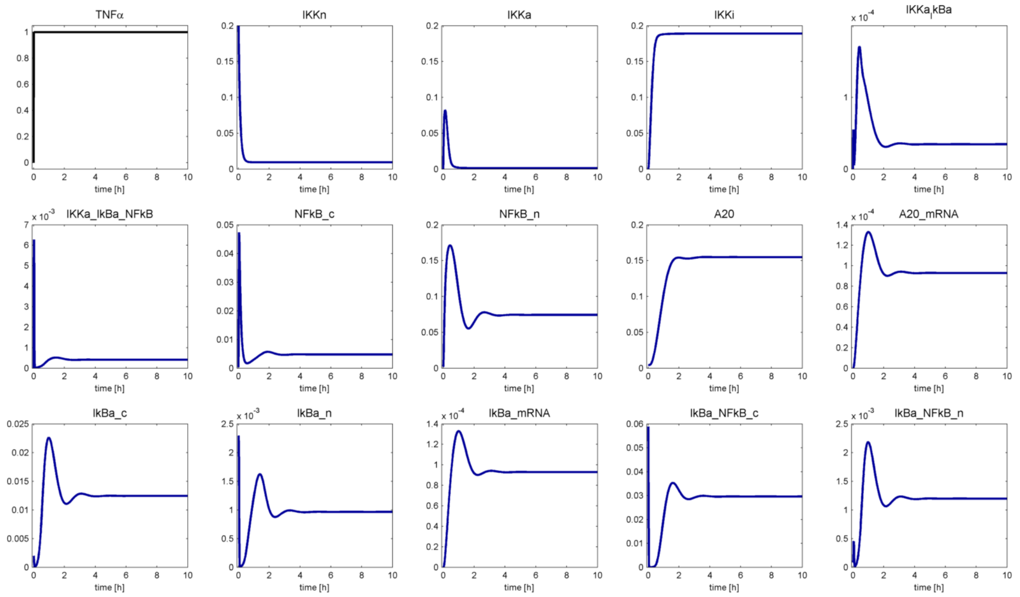

Figure S4. Simulation results of the NF-κB module from Schlatter et al. (2011). This module is based on a previously published model (Lipniacki et al., 2004)

Table S2. Parameters of the NF-κB model. Notation of all parameters of the NF-κB model from Schlatter et al. (2011) with their value and a description.

parameter	value	description
k_{Nk1}	9	activation of IKK by TNFα
k_{Nk2}	360	inhibition of IKK by TNFα and A20
k_{Nk3}	5.4	spontaneous inhibition of IKK
k_{Nkprod}	0.09	constitutive production of IKKn
k_{Nkdeg}	0.45	degradation of IKK species
k_{Na1}	1800	inhibition of NF-κB via binding of IκBα
k_{Na2}	720	complex formation of IκBα and IKKa
k_{Na3}	3600	binding of IKKa to the IκBα_NF-κB complex
k_{Nt1}	360	liberation of IKKa
k_{Nt2}	360	liberation of IKK and NF-κB
k_{Nc1}	0.0018	NF-κB-induced synthesis of A20 mRNA
k_{Nc1a}	0.0018	NF-κB-induced synthesis of IκBα mRNA
k_{Nc2}	0	constitutive synthesis of A20 mRNA
k_{Nc2a}	0	constitutive synthesis of IκBα mRNA
k_{Nc3}	1.44	degradation of A20 mRNA
k_{Nc3a}	1.44	degradation of IκBα mRNA
k_{Nc4}	1800	synthesis of A20
k_{Nc4a}	1800	synthesis of IκBα
k_{Nc5}	1.08	degradation of A20
k_{Nc5a}	0.36	degradation of cytosolic IκBα
k_{Nc6a}	0.072	liberation of NF-κB via degradation of IκBα
k_{Ni1}	9	translocation of cytosolic NF-κB to the nucleus
k_{Ni1a}	3.6	translocation of cytosolic IκBα to the nucleus
k_{Ne1a}	1.8	translocation of nuclear IκBα to the cytosol
k_{Ne2a}	36	translocation of nuclear IκBα_NF-κB to the cytosol
k_V	3	volume ratio of cytosol to nucleus

4.1 Model Equations

$$\dot{x}_{\text{A20}} = k_{\text{Nc4}} \cdot x_{\text{A20mRNA}} - k_{\text{Nc5}} \cdot x_{\text{A20}} \tag{S2}$$

$$\dot{x}_{\text{A20mRNA}} = k_{\text{Nc1}} \cdot x_{\text{NF}\kappa\text{B\_n}} + k_{\text{Nc2}} - k_{\text{Nc3}} \cdot x_{\text{A20mRNA}} \tag{S3}$$

$$\dot{x}_{\text{NF}\kappa\text{B\_n}} = k_{\text{Ni1}} \cdot k_V \cdot x_{\text{NF}\kappa\text{B\_c}} - k_{\text{Na1}} \cdot x_{\text{NF}\kappa\text{B\_n}} \tag{S4}$$

$$\begin{aligned}\dot{x}_{\text{NF}\kappa\text{B\_c}} &= k_{\text{Nc6a}} \cdot x_{\text{I}\kappa\text{B}\alpha\text{\_NF}\kappa\text{B\_c}} + k_{\text{Nt2}} \cdot x_{\text{IKKa\_I}\kappa\text{B}\alpha\text{\_NF}\kappa\text{B}} \\ &\quad - k_{\text{Ni1}} \cdot x_{\text{NF}\kappa\text{B\_c}} - k_{\text{Na1}} \cdot x_{\text{NF}\kappa\text{B\_c}} \cdot x_{\text{I}\kappa\text{B}\alpha\text{\_c}}\end{aligned} \tag{S5}$$

$$\dot{x}_{\text{I}\kappa\text{B}\alpha\text{mRNA}} = k_{\text{Nc1a}} \cdot x_{\text{NF}\kappa\text{B\_n}} + k_{\text{Nc2a}} - k_{\text{Nc3a}} \cdot x_{\text{I}\kappa\text{B}\alpha\text{mRNA}} \tag{S6}$$

$$\begin{aligned}\dot{x}_{\text{I}\kappa\text{B}\alpha\text{\_n}} &= k_{\text{Ni1a}} \cdot k_V \cdot x_{\text{I}\kappa\text{B}\alpha\text{\_c}} - k_{\text{Ne1a}} \cdot k_V \cdot x_{\text{I}\kappa\text{B}\alpha\text{\_n}} \\ &\quad - k_{\text{Na1}} \cdot x_{\text{NF}\kappa\text{B\_n}} \cdot x_{\text{I}\kappa\text{B}\alpha\text{\_n}}\end{aligned} \tag{S7}$$

$$\begin{aligned}\dot{x}_{\text{I}\kappa\text{B}\alpha\text{\_c}} &= k_{\text{Nc4a}} \cdot x_{\text{I}\kappa\text{B}\alpha\text{mRNA}} + k_{\text{Ne1a}} \cdot x_{\text{I}\kappa\text{B}\alpha\text{\_n}} - k_{\text{Ni1a}} \cdot x_{\text{I}\kappa\text{B}\alpha\text{\_c}} \\ &\quad - k_{\text{Na1}} \cdot x_{\text{I}\kappa\text{B}\alpha\text{\_c}} \cdot x_{\text{NF}\kappa\text{B\_c}} - k_{\text{Na2}} \cdot x_{\text{IKKa}} \cdot x_{\text{I}\kappa\text{B}\alpha\text{\_c}} \\ &\quad - k_{\text{Nc5a}} \cdot x_{\text{I}\kappa\text{B}\alpha\text{\_c}}\end{aligned} \tag{S8}$$

$$\begin{aligned}\dot{x}_{\text{I}\kappa\text{B}\alpha\text{NF}\kappa\text{B\_c}} &= k_{\text{Na1}} \cdot x_{\text{NF}\kappa\text{B\_c}} \cdot x_{\text{I}\kappa\text{B}\alpha\text{\_c}} + k_{\text{Ne2a}} \cdot x_{\text{I}\kappa\text{B}\alpha\text{NF}\kappa\text{B\_n}} \\ &\quad - k_{\text{Nc6a}} \cdot x_{\text{I}\kappa\text{B}\alpha\text{NF}\kappa\text{B\_c}} - k_{\text{Na3}} \cdot x_{\text{IKKa}} \cdot x_{\text{I}\kappa\text{B}\alpha\text{NF}\kappa\text{B\_c}}\end{aligned} \tag{S9}$$

$$\dot{x}_{\text{I}\kappa\text{B}\alpha\text{NF}\kappa\text{B\_n}} = k_{\text{Na1}} \cdot x_{\text{I}\kappa\text{B}\alpha\text{\_n}} \cdot x_{\text{NF}\kappa\text{B\_n}} - k_{\text{Ne2a}} \cdot k_V \cdot x_{\text{I}\kappa\text{B}\alpha\text{NF}\kappa\text{B\_n}} \tag{S10}$$

$$\begin{aligned}\dot{x}_{\text{IKKa}} &= k_{\text{Nk1}} \cdot x_{\text{IKKn}} \cdot x_{\text{TNF}\alpha} - k_{\text{Nk2}} \cdot x_{\text{IKKa}} \cdot x_{\text{A20}} \cdot x_{\text{TNF}\alpha} \\ &\quad - k_{\text{Nk3}} \cdot x_{\text{IKKa}} - k_{\text{Nkdeg}} \cdot x_{\text{IKKa}} - k_{\text{Na2}} \cdot x_{\text{IKKa}} \cdot x_{\text{I}\kappa\text{B}\alpha\text{\_c}} \\ &\quad - k_{\text{Na3}} \cdot x_{\text{IKKa}} \cdot x_{\text{I}\kappa\text{B}\alpha\text{NF}\kappa\text{B\_c}} + k_{\text{Nt1}} \cdot x_{\text{IKKaI}\kappa\text{B}\alpha} \\ &\quad + k_{\text{Nt2}} \cdot x_{\text{IKKaI}\kappa\text{B}\alpha\text{NF}\kappa\text{B}}\end{aligned} \tag{S11}$$

$$\dot{x}_{\text{IKKn}} = k_{\text{Nkprod}} - k_{\text{Nk1}} \cdot x_{\text{IKKn}} \cdot x_{\text{TNF}\alpha} - k_{\text{Nkdeg}} \cdot x_{\text{IKKn}} \tag{S12}$$

$$\dot{x}_{\text{IKKi}} = k_{\text{Nk2}} \cdot x_{\text{IKKa}} \cdot x_{\text{A20}} \cdot x_{\text{TNF}\alpha} + k_{\text{Nk3}} \cdot x_{\text{IKKa}} - k_{\text{Nkdeg}} \cdot x_{\text{IKKi}} \tag{S13}$$

$$\dot{x}_{\text{IKKaI}\kappa\text{B}\alpha} = k_{\text{Na2}} \cdot x_{\text{IKKa}} \cdot x_{\text{I}\kappa\text{B}\alpha\text{\_c}} - k_{\text{Nt1}} \cdot x_{\text{IKKaI}\kappa\text{B}\alpha} \tag{S14}$$

$$\dot{x}_{\text{IKKaI}\kappa\text{B}\alpha\text{NF}\kappa\text{B}} = k_{\text{Na3}} \cdot x_{\text{IKKa}} \cdot x_{\text{I}\kappa\text{B}\alpha\text{NF}\kappa\text{B\_c}} - k_{\text{Nt2}} \cdot x_{\text{IKKaI}\kappa\text{B}\alpha\text{NF}\kappa\text{B}} \tag{S15}$$

4.2 Parameter Variations

We analyzed the influence of parameter variations in the range of two orders of magnitude on the dynamics of nuclear NF-κB, which is the active form inducing transcription of respective target genes (Figure S5). From the 25 parameters analyzed, 3 parameters, i.e. Nc2, Nc2a, and Nt1, have no or minor influence on the dynamics of NF-κB. Many parameters only influence the dynamics of NF-κB when they are varied in one direction. Increasing the value of the parameters Nk3, Nkdeg, and Na2 influences the time course of NF-κB, whereas decreasing the value of these parameters has no influence. Furthermore, often only a change of the parameter value by two orders of magnitude has influence on the dynamics of NF-κB, i.e. a 100-fold increase of Nc5a, Nc6a, and Ne1a as well as a 100-fold decrease of Na1 and Ne2a influences the time course of NF-κB, whereas smaller parameter changes have no influence. This

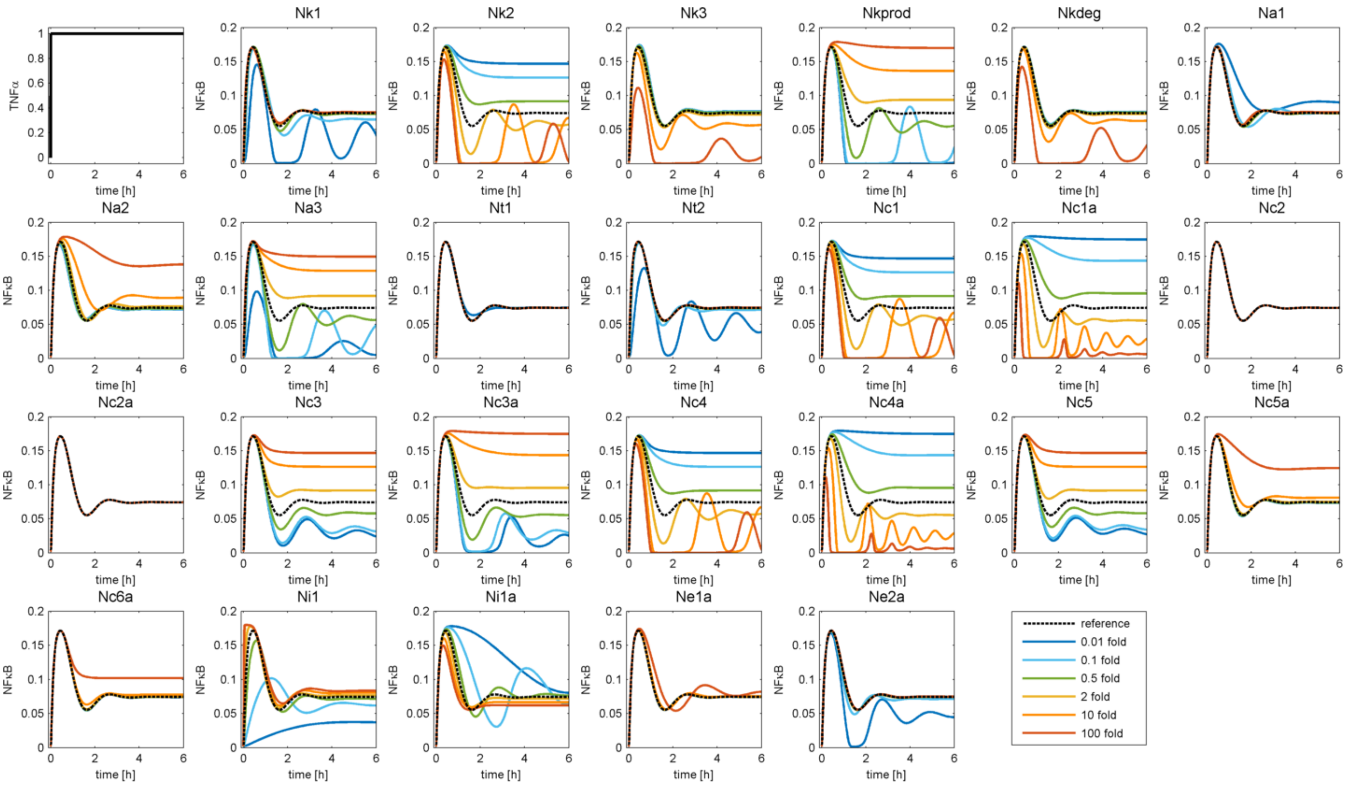

Figure S5. Influence of parameter variations on NF-κB. All parameters of the NF-κB module were varied in the range of two orders of magnitude and the time course of nuclear NF-κB was simulated for the first 6 hours following TNFα stimulation.

indicates that the parameter are not well adjusted and the model ist too complex, especially when only limited experimental data is available. The focus here lays on description of NF-κB activation and target gene expression and, therefore, the complex formations of IKK, IκBα and NF-κB as well as transport steps between the cytosol and the nucleus, which occure very fast, can be neglected.

4.3 Model Reduction

The NF-κB module was reduced to both decrease the number of species and parameters.

- The separation of IKK species in neutral and deactivated form was abolished and only the activating phosphorylation step of IKK by TNFα was modeled, as also implemented by others, e.g. Pinna et al. (2012).
- Furthermore, the activated form IKKp induces phosphorylation and degradation of IκBα and, thus, activation of NF-κB, but the complexes IKKa_IκBα and IKKa_IκBα_NF-κB are negelcted.
- It was assumed that the phosphorylation/dephosphorylation step is pivotal for IKK kinetics and, thus, synthesis and degradation is neglected.
- Furthermore, it was assumed that the transport steps between the cytosol and the nucleus occure very fast and can also be neglected.
- The number of parameters was further decreased by assuming that mRNA synthesis and degradation of both A20 and IκBα mRNA occure comparably fast.

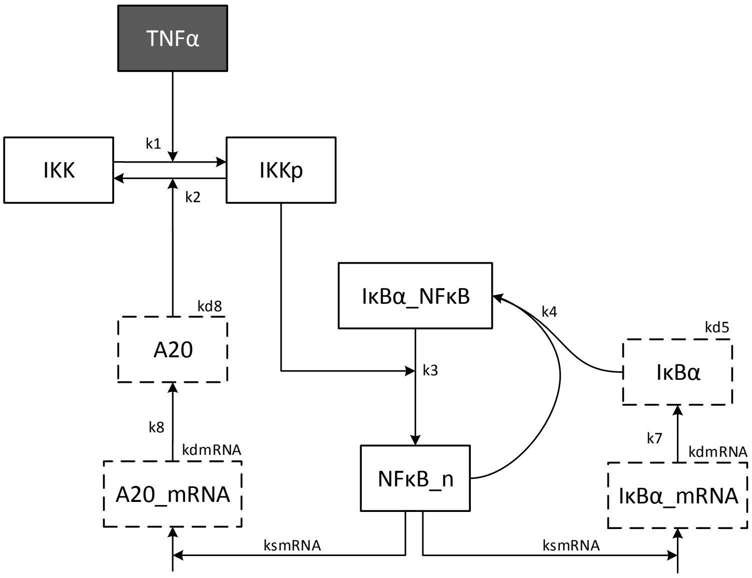

Figure S6. Reduced NF-κB module. Structure of the reduced NF-κB module. The model now has 8 species and 10 parameters. Input of the model is TNFα. The model is based on ordinary differential equations (ODEs) and mass action kinetics. Degradation of species is indicated by boxes with dashed borders

The reduced model scheme is displayed in Figure S6. The model, now, has only 8 instead of 14 species and 10 instead of 26 parameters. All species and parameters of the reduced model are liste in Table S3 and Table S4, respectively.

4.4 Model Equations

$$\dot{x}_{\mathrm{IKK}} = -k_1 \cdot x_{\mathrm{IKK}} \cdot x_{\mathrm{TNF}\alpha} + k_2 \cdot x_{\mathrm{IKKp}} \cdot x_{\mathrm{A20}} \tag{S16}$$

$$\dot{x}_{\mathrm{IKKp}} = +k_1 \cdot x_{\mathrm{IKK}} \cdot x_{\mathrm{TNF}\alpha} - k_2 \cdot x_{\mathrm{IKKp}} \cdot x_{\mathrm{A20}} \tag{S17}$$

$$\dot{x}_{\mathrm{NF\kappa B}} = k_3 \cdot x_{\mathrm{IKKp}} \cdot x_{\mathrm{I\kappa B\alpha NF\kappa B}} - k_4 \cdot x_{\mathrm{NF\kappa B}} \cdot x_{\mathrm{I\kappa B\alpha}} \tag{S18}$$

$$\dot{x}_{\mathrm{I\kappa B\alpha}} = -k_4 \cdot x_{\mathrm{NF\kappa B}} \cdot x_{\mathrm{I\kappa B\alpha}} + k_7 \cdot x_{\mathrm{I\kappa B\alpha mRNA}} - k_{d5} \cdot x_{\mathrm{I\kappa B\alpha}} \tag{S19}$$

$$\dot{x}_{\mathrm{I\kappa B\alpha NF\kappa B}} = -k_3 \cdot x_{\mathrm{IKKp}} \cdot x_{\mathrm{I\kappa B\alpha NF\kappa B}} + k_4 \cdot x_{\mathrm{NF\kappa B}} \cdot x_{\mathrm{I\kappa B\alpha}} \tag{S20}$$

$$\dot{x}_{\mathrm{I\kappa B\alpha mRNA}} = k_{smrna} \cdot x_{\mathrm{NF\kappa B}} - k_{dmrna} \cdot x_{\mathrm{I\kappa B\alpha mRNA}} \tag{S21}$$

$$\dot{x}_{\mathrm{A20mRNA}} = k_{smrna} \cdot x_{\mathrm{NF\kappa B}} - k_{dmrna} \cdot x_{\mathrm{A20mRNA}} \tag{S22}$$

$$\dot{x}_{\mathrm{A20}} = k_8 \cdot x_{\mathrm{A20mRNA}} - k_{d8} \cdot x_{\mathrm{A20}} \tag{S23}$$

Table S3. Species of the reduced model. Notation of all species of the reduced NF-κB module (Figure S6) with their official symbol as well as full name and gene ID according to the NCBI gene data base or description.

species	official symbol	official full name / description	Gene ID
IKK	Chuk<br>Ikbkb<br>Ikbkg	IKK complex comprising IKKα/Chuk (conserved helix-loop-helix ubiquitous kinase), IKKβ/Ikbkb (inhibitor of kappaB kinase beta) and IKKγ/Ikbkg (inhibitor of kappaB kinase gamma)	12675<br>16150<br>16151
IKKp	-	phosphorylated, active form of IKK	-
NFκB	Rela	v-rel reticuloendotheliosis viral oncogene homolog A (avian)	19697
IκBα	Nfkbia	nuclear factor of kappa light polypeptide gene enhancer in B cells inhibitor, alpha	18035
IκBα_NFκB	-	complex of NF-κB and IκBα (inactivated NF-κB)	-
IκBα_mRNA	-	mRNA encoding IκBα	-
A20_mRNA	-	mRNA encoding A20	-
A20	Tnfaip3	tumor necrosis factor, alpha-induced protein 3	21929

Table S4. Parameter values of the reduced model. Notation of all parameters of the reduced NF-κB module (Figure S6) with their value and a description.

parameter	value	description
k_1	4.5	activation of IKK by TNFα
k_2	4150	inhibition of IKK by A20
k_3	900	activation of NF-κB by phosphorylation and degradation of IκBα
k_4	150	deactivation of NF-κB via binding of IκBα
k_7	900	IκBα protein synthesis
k_8	2000	A20 protein synthesis
k_{smrna}	5E-03	mRNA synthesis
k_{dmrna}	1.44	mRNA degradation
k_{d5}	2.4	degradation of IκBα
k_{d8}	1.4	degradation of A20

4.5 Parameterization and Initial Conditions

Parameters were manually adjusted to reproduce the behavior of the original model as precisely as possible, expecially the dynamics of NF-κB. All parameter values are listed in Table S4. Some species of the original model directly correspond to species of the reduced model, i.e. IκBα mRNA, A20 mRNA, A20, and IKKp that was formerly named IKKa. But some trajectories of the original model needed to be summed up to obtain comparable time courses, relating to the assumptions that werde made for model reduction: The neutral (IKKn) and deactivated (IKKi) form of IKK were summarized to one dephosphorylated form of IKK. The cytosolic and nuclear species were also summarized, whereby the factor of cytosolic to nuclear volume (k_V) needed to be considered. The complexes of IKKa with IκBα and/or NF-κB were not explicitely modeled and, thus, their trajectories were added to the single species, respectively. Thereby, the following correlations of species from the original ($x_{i,\text{org}}$) and reduced ($x_{i,\text{red}}$) model were derived:

$$x_{\text{IKK,red}} = x_{\text{IKKn,org}} + x_{\text{IKKi,org}} \tag{S24}$$

$$x_{\text{IKKp,red}} = x_{\text{IKKa,org}} \tag{S25}$$

$$x_{\text{NF}\kappa\text{B,red}} = x_{\text{NF}\kappa\text{B\_c,org}} + 1/k_V \cdot x_{\text{NF}\kappa\text{B\_n,org}} \tag{S26}$$

$$x_{\text{I}\kappa\text{B}\alpha\text{,red}} = x_{\text{I}\kappa\text{B}\alpha\text{\_c,org}} + 1/k_V \cdot x_{\text{I}\kappa\text{B}\alpha\text{\_n,org}} + x_{\text{IKKaI}\kappa\text{B}\alpha\text{,org}} \tag{S27}$$

$$x_{\text{I}\kappa\text{B}\alpha\text{NF}\kappa\text{B,red}} = x_{\text{I}\kappa\text{B}\alpha\text{NF}\kappa\text{B\_c,org}} + 1/k_V \cdot x_{\text{I}\kappa\text{B}\alpha\text{NF}\kappa\text{B\_n,org}} + x_{\text{IKKaI}\kappa\text{B}\alpha\text{NF}\kappa\text{B,org}} \tag{S28}$$

$$x_{\text{I}\kappa\text{B}\alpha\text{mRNA,red}} = x_{\text{I}\kappa\text{B}\alpha\text{mRNA,org}} \tag{S29}$$

$$x_{\text{A20mRNA,red}} = x_{\text{A20mRNA,org}} \tag{S30}$$

$$x_{\text{A20,red}} = x_{\text{A20,org}} \tag{S31}$$

Initial conditions were calculated accordingly and under the additional constraint that the system without stimulation ($x_{\text{TNF}\alpha} = 0$) is at steady state ($\dot{x}_i$ = 0, with $x_i > 0$) for all parameter values $k_i > 0$:

$$x_{\text{IKK,red}}(0) = x_{\text{IKKn,org}}(0) + x_{\text{IKKi,org}}(0) = 0.2 \tag{S32}$$

$$x_{\text{IKKp,red}}(0) = x_{\text{IKKa,org}}(0) = 0 \tag{S33}$$

$$x_{i,\text{mRNA,red}}(0) = x_{i,\text{mRNA,org}}(0) = 0 \tag{S34}$$

$$\begin{aligned} \dot{x}_{\text{A20}} &= k_8 \cdot x_{\text{A20mRNA}} - k_{d8} \cdot x_{\text{A20}} = 0 \\ &\quad \text{with } x_{\text{A20mRNA,red}}(0) = 0 \;\rightarrow\; x_{\text{A20,red}}(0) = 0 \end{aligned} \tag{S35}$$

$$\begin{aligned} \dot{x}_{i,\text{mRNA}} &= k_{smrna} \cdot x_{\text{NF}\kappa\text{B}} - k_{dmrna} \cdot x_{i,\text{mRNA}} = 0 \\ &\quad \text{with } x_{i,\text{mRNA,red}}(0) = 0 \;\rightarrow\; x_{\text{NF}\kappa\text{B,red}}(0) = 0 \end{aligned} \tag{S36}$$

$$\begin{aligned} \dot{x}_{\text{I}\kappa\text{B}\alpha} &= -k_4 \cdot x_{\text{NF}\kappa\text{B}} \cdot x_{\text{I}\kappa\text{B}\alpha} + k_7 \cdot x_{\text{I}\kappa\text{B}\alpha\text{mRNA}} - k_{d5} \cdot x_{\text{I}\kappa\text{B}\alpha} = 0 \\ &\quad \text{with } x_{\text{I}\kappa\text{B}\alpha\text{mRNA,red}}(0) = 0 \;\rightarrow\; x_{\text{I}\kappa\text{B}\alpha\text{,red}}(0) = 0 \end{aligned} \tag{S37}$$

$$\begin{aligned} x_{\text{I}\kappa\text{B}\alpha\text{NF}\kappa\text{B,red}}(0) &= x_{\text{I}\kappa\text{B}\alpha\text{NF}\kappa\text{B\_c,org}}(0) + 1/k_V \cdot x_{\text{I}\kappa\text{B}\alpha\text{NF}\kappa\text{B\_n,org}}(0) \\ &\quad + x_{\text{IKKaI}\kappa\text{B}}(0) + x_{\text{NF}\kappa\text{B\_c,org}}(0) + 1/k_V \cdot x_{\text{NF}\kappa\text{B\_n,org}}(0) \\ &\quad + x_{\text{I}\kappa\text{B}\alpha\text{\_c,org}}(0) + 1/k_V \cdot x_{\text{I}\kappa\text{B}\alpha\text{\_n,org}}(0) + x_{\text{IKKaI}\kappa\text{B}\alpha\text{,org}}(0) \\ &= 0.0625 \end{aligned} \tag{S38}$$

The simulation results of the reduced model in comparison to the original trajectories, calculated as described above, are shown in Figure S7. As it can be seen, the time courses are in good comparison. Especially the trajectory of NFκB from the reduced model only minimally differs from the original model. The reduced model, therefor, is now suitable to study the dynamics of NFκB activation and target gene expression.

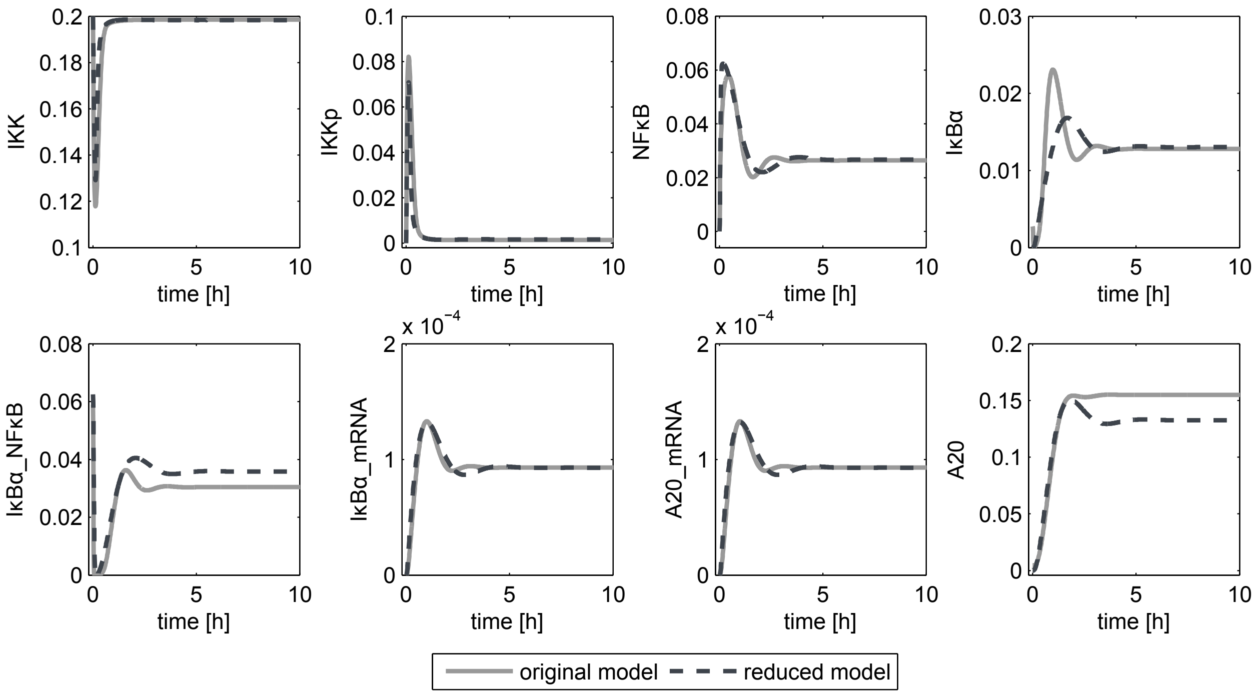

Figure S7. Comparison of the simulated time courses of the original and reduced NF-κB module.

5 FURTHER SUPPLEMENTARY MATERIAL

Data Sheet 1. Transcript expression data of IL-1β and TNFα-stimulated hepatocytes. Mean expression values of measured mRNAs from primary murine hepatocytes stimulated with IL-1β (20 ng/ml) or TNFα (25 ng/ml) for 1, 4, 6, 18 and 30 h normalized on untreated controls with standard deviation and p-values for regulation compared to untreated controls as well as the official full name and gene ID as given in the NCBI gene database and the Applied Biosystems assay number.

Data Sheet 2. Transcript expression data of hepatocytes stimulated with supernatant from BMDMs. BMDMs were stimulated with 100 ng/ml LPS for 24 h or left untreated as control and supernatants were collected. Primary murine hepatocytes were cultured on collagen and, after starvation, incubated with DMEM medium for 4 h that was also used for cultivation of BMDMs. Afterwards, hepatocytes were stimulated with conditioned or unconditioned supernatant from BMDM for 12 h and with FasL (50 ng/ml) for further 6 h. Data is analyzed using the ddCT method (Livak and Schmittgen, 2001) and normalized on controls. Mean expression values of measured mRNAs with standard deviation and p-values for regulation compared to controls (hepatocytes treated with DMEM only) are shown as well as the official full name and gene ID as given in the NCBI gene database and the Applied Biosystems assay number.

REFERENCES

Lipniacki, T., Paszek, P., Brasier, A. R. A. R., Luxon, B., and Kimmel, M. (2004). Mathematical model of NF-kappaB regulatory module. *Journal of theoretical biology* 228, 195–215. doi:10.1016/j.jtbi.2004.01.001

Livak, K. J. and Schmittgen, T. D. (2001). Analysis of relative gene expression data using real-time quantitative PCR and the 2(-Delta Delta C(T)) Method. *Methods (San Diego, Calif.)* 25, 402–8. doi:10.1006/meth.2001.1262

Lutz, A., Sanwald, J., Thomas, M., Feuer, R., Sawodny, O., Ederer, M., et al. (2014). Interleukin-1β enhances FasL-induced caspase-3/-7 activity without increasing apoptosis in primary mouse hepatocytes. *PloS one* 9, e115603. doi:10.1371/journal.pone.0115603

Pinna, F., Sahle, S., Beuke, K., Bissinger, M., Tuncay, S., D'Alessandro, L. A., et al. (2012). A systems biology study on NF??B signaling in primary mouse hepatocytes. *Frontiers in Physiology* 3 DEC, 1–14. doi:10.3389/fphys.2012.00466

Schlatter, R., Schmich, K., Lutz, A., Trefzger, J., Sawodny, O., Ederer, M., et al. (2011). Modeling the TNFα-Induced Apoptosis Pathway in Hepatocytes. *PLoS ONE* 6, e18646. doi:10.1371/journal.pone.0018646
